# A new ergasilid copepod from lates perches in East Africa: morphology, phylogenetics, and genetic structure of *Ergasilus ereimia* sp. nov.

**DOI:** 10.3389/fvets.2025.1699263

**Published:** 2026-02-02

**Authors:** Ferre Vandenberg, Nikol Kmentová, Hiram Karanja, Maarten Van Steenberge, Nathan Vranken, Maarten P. M. Vanhove, Kelly J. M. Thys

**Affiliations:** 1 Research Group Zoology: Biodiversity and Toxicology, Centre for Environmental Sciences, Hasselt University, Diepenbeek, Belgium; 2 Royal Belgian Institute of Natural Sciences, Operational Directorates Natural Environment, Taxonomy and Phylogeny, Brussels, Belgium; 3 Kenya Marine and Fisheries Research Institute, Mombasa, Kenya; 4 Section Vertebrates, Biology Department, Royal Museum for Central Africa, Tervuren, Belgium

**Keywords:** African Ergasilidae, biodiversity, Cyclopoida, Lake Turkana, Lake Albert, Latidae, parasitic copepods

## Abstract

Copepods are widely distributed across marine and freshwater environments and are often praised for their immense taxonomic and functional diversity. However, relatively little is known about parasitic copepods, particularly regarding their phylogenetic relationships. This study investigates the morphology and phylogenetic positioning of a proposed new species of parasitic copepods described as *Ergasilus ereimia* sp. nov. (Ergasilidae). The ectoparasitic female copepods (1,645 specimens) were obtained by performing parasitological screening of ethanol-preserved gills of lates perches from Lake Turkana (Kenya; 4 specimens of *Lates niloticus*, 6 specimens of *Lates longispinis*) and Lake Albert (Uganda; 5 specimens of *L. niloticus*) in East Africa. Light and confocal microscopy were used to conduct the morphological characterisation and to determine the spine-seta formula of the parasitic females. A differential diagnosis was conducted with all 18 other formally described species of *Ergasilus* from the African continent, which revealed *E. ereimia* sp. nov. to have a unique combination of morphological traits and a unique spine-seta formula. A phylogenetic tree was constructed using the concatenated partial sequences of the 18S and 28S ribosomal DNA (rDNA) genes. We hypothesised that *E. ereimia* sp. nov. would belong to the same clade as the other continental African ergasilids. This proved to be correct, and this taxon forms a well-supported sister clade to the other continental African species of *Ergasilus* with available sequence data. The intra- and interspecific model-corrected genetic distances were calculated based on the fragments of the 18S rDNA (average of 0.001 and 0.031 respectively) and 28S rDNA (average of 0.001 and 0.154 respectively) genetic markers, as well as on a fragment (1,122 bp) of the cytochrome *c* oxidase subunit I (COI) mitochondrial DNA (mtDNA) sequences (intraspecific average of 0.019), all of which further support the designation of a novel species of ergasilid copepods. A Neighbour Joining haplotype network based on the fragment of COI mtDNA showed ongoing diversification between the populations of *E. ereimia* sp. nov. from Lake Turkana and Lake Albert, in addition to the observed continuous intraspecific morphological variation in size and pigmentation.

## 1 Introduction

Copepods are widely distributed globally across marine and freshwater ecosystems, exhibiting extraordinary taxonomic and functional diversity (1). Parasitism has evolved independently in multiple lineages of free-living copepods (2), with *Ergasilus* von Nordmann 1832 being one of the most species-rich parasitic copepod genera, comprising 197 known species worldwide (3, 4). However, *Ergasilus* is considered to be a polyphyletic taxon (3, 5–7). Members of this genus belong to Ergasilidae (order Cyclopoida), where the copepodid stages, adult males, and pre-mated adult females exhibit a semi-planktonic lifestyle, while only the post-mated adult females parasitise on fish (8, 9). Most ergasilid species have a low host specificity (meaning they can infect a wide range of host species) (10), sometimes even infecting hosts of different fish families (11). The adult male copepods do not engage with the host and typically die after mating (12).

**Figure 1:**
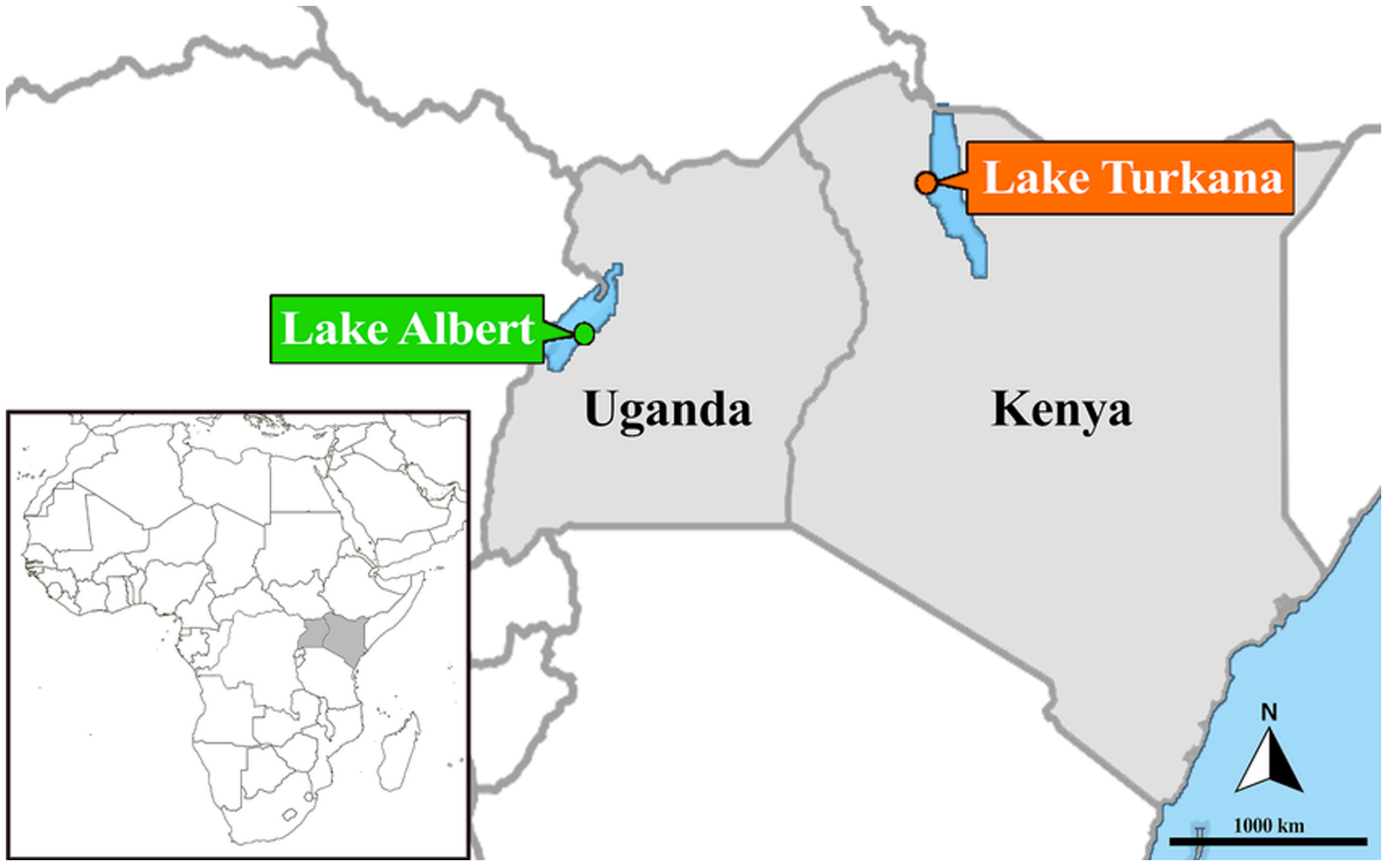
Map of the sampling localities of Nile perches at Ferguson’s Gulf of Lake Turkana in Kenya (orange; 03°30′40.74”N, 35°54′53.00″E) and at Kaiso landing site at Lake Albert in Uganda (green; 01°31′59.40”N, 30°57′59.95″E). Countries in which the samples were taken are shaded.

The Nile perch, *Lates niloticus* (Linnaeus 1782) (Actinopterygii, Carangaria *incertae sedis*, Latidae) (4), is a freshwater bony fish of immense importance as both a food source and a source of income for communities in East Africa (13). It is distributed across many major river basins of sub-Saharan Africa, including the Nile, Congo, Chad, Niger, Senegal, and Volta (13). In Eastern Africa, the Nile perch is native to Lakes Turkana and Albert (14). Lake Turkana is an endorheic and alkaline basin located in North Eastern Kenya and Southern Ethiopia (Figure 1), with approximately 90% of its inflow coming from the Omo River (15). Lake Albert is located on the border of Uganda and the Democratic Republic of Congo (Figure 1) and receives inflow from the Victoria Nile (from Lake Victoria via Lake Kyoga) and the Semliki River (from Lake Edward). The fish fauna of Lake Albert is separated from that of these lakes by a series of falls, which drain north through the Albert Nile. Notably, Lake Turkana harbours two species of lates perches, *L. niloticus* and *Lates longispinis* (Worthington 1932), both of which are native (17), with the nominal species *L. longispinis* being endemic to the lake (16). The neighbouring Lake Albert harbours *L. niloticus* and the endemic nominal species *Lates macrophthalmus* Worthington 1929 (18). However, taxonomic uncertainties persist around the status of lates perches in these two lakes (19, 20).

Due to a paucity of research efforts, the true species diversity and phylogenetic relationships of Ergasilidae in Africa remain to be discovered (3). In the last decade, only five new African ergasilids were described by Míč et al. (3) and van der Spuy et al. (21), and the only known ergasilids to infect *L. niloticus* are *Ergasilus kandti* van Douwe 1912 (22–26), and *Ergasilus latus* Fryer 1960 (28). The study of parasitic copepods (and of parasites in general) is of crucial importance since they can significantly affect the growth, fecundity, and longevity of their host (29). Ergasilid copepods feed on the gill tissue, mucus, and blood of the host (29), and their attachment and feeding activities have been observed to be responsible for gill inflammation, necrosis, high levels of mucus production, and secondary infections (30, 31). Outbreaks of diseases caused by these parasites can even result in substantial host mortality, as documented by Paperna (32), who reported heavy infections of *Ergasilus lizae* Krøyer 1863 on cultured *Mugil cephalus* Linnaeus 1758 in Israel.

The aim of this study was to (1) explore the diversity of ergasilid copepods infecting lates perches (*L. niloticus* and *L. longispinis*) in Lakes Turkana and Albert, and to (2) investigate their morphological and genetic variability within and between the lakes. We Hypothesised that the proposed novel species *Ergasilus ereimia* sp. nov. would belong to the same clade as the other continental African ergasilids.

## 2 Materials and methods

### 2.1 Sampling of lates perches and their gill parasites

Fresh specimens of lates perches were obtained from Lake Turkana using a purse seine and gillnet at Ferguson’s Gulf, Kenya (03°30′40.74”N, 35°54′53″E; September 2022) by H. Karanja and collaborators at Kenya Marine and Fisheries Research Institute (KMFRI, Turkana station), and from Lake Albert at Kaiso landing site, Uganda (01°31′59”N, 30°57′60″E; April 2019) within the Belgian Science Policy (BELSPO)-funded Brain Project: Human impacts on ecosystem health and resources of Lake Edward (HIPE; BR/154/A1/HIPE). The sampling permit for Lake Turkana was granted by Kenya’s National Commission for Science, Technology & Innovation (NACOSTI) under license NACOSTI/P/22/20570. A map of the sampling localities is depicted in Figure 1. Host specimens were morphologically identified based on their diagnostic characters, as outlined by Worthington (16, 18). Gill pairs of four specimens of *L. niloticus* and six specimens of *L. longispinis* were sampled in Lake Turkana. Five gills from the right side of *L. niloticus* were collected from Lake Albert (Table 1). All gills, preserved in absolute ethanol, were screened for the presence of ectoparasites using a Leica EZ4 stereomicroscope and entomological needles.

**Table 1:** Overview of the number and size range of host specimens of lates perches, the number of collected gill parasites, and the copepod infection parameters (prevalence, the percentage of hosts infected with copepods; and mean infection intensity, the mean number of copepods per infected host) for Lakes Turkana and Albert.

Water body	Host species	*n* hosts	Host size range (mm)	Parasite taxon	*n* parasite	Prevalence (%)	Mean infection intensity
Lake Turkana	*Lates niloticus*	4	383–540	Copepoda	658	100	164.5
	*Lates longispinis*	6	243–311	Copepoda	937	100	156.2
Lake Albert	*Lates niloticus*	5	360–435	Copepoda	50	80	10.0

With *n*, the sample size.

Parasite infection parameters were calculated separately for the different host species from Lakes Turkana and Albert (Table 1). Prevalence (P) represents the relative number of infected hosts, calculated by dividing the number of infected hosts by the number of screened hosts. The mean infection intensity (MI) reflects the mean number of parasites per infected host, calculated by dividing the number of parasite specimens by the number of infected hosts (33).

### 2.2 Morphological identification of the parasitic copepods

The soft tissues of the copepod specimens were digested during the first steps of the DNA extraction (see 2.4 DNA extractions, PCR, and Sanger sequencing), leaving the carapace intact for morphological identification using light microscopy. The carapaces and undigested copepod specimens were stained with Congo Red (1 mg/mL aqueous solution) for at least 24 h. Following a 30-min rinse in deionised water, the specimens were mounted with glycerol on microscopy slides (5, 34), which were then sealed using Glyceel (35).

A Leica DM 2500 LED light microscope, mounted with a Leica DFC450 C camera connected to the LAS X software, was used for the imaging (200×, 400×, and 1,000× magnification) and measuring of the specimens (200× magnification). For light microscopy, 48 specimens were mounted on regular slides, of which 45 specimens were stained. Digital drawings were made in Affinity Photo v2.5.6 using the XP-Pen Artist 15.6 Pro pen display.

For the acquisition of three-dimensional scans with confocal laser microscopy, a ZEISS LSM900 Airyscan 2 and the Zen Blue software were used with an excitation wavelength of 561 nm (mCherry dye); an emission wavelength in the range of 565–700 nm; a pinhole size of 1 airy unit (AU); a laser intensity of 0.2%; 200× magnification; resolution of 319.5 × 319.5 μm; bidirectional scanning; 4× averaging; 8 bits per pixel; a pixel time of 7; 10–15 tiles (depending on the orientation of the specimen) and an optimal interval in the Z-stack. For confocal microscopy, 12 specimens were mounted on regular slides and 10 specimens on concave slides. Of these specimens, 16 were stained. The soft tissues of the specimens were typically not digested for confocal microscopy, with the exception of one specimen. This difference in sample preparation did not affect confocal image acquisition.

The obtained light and confocal microscopic images were used to investigate the morphological characteristics of the copepods, as well as to ascertain the spine-seta formula of the swimming legs. This formula depicts the number of spines and setae present on the segments of the swimming legs, and is used as a diagnostic feature for the identification of copepod species (36). Following Schlebush (37), setae were distinguished as elongate structures that maintain a relatively uniform width along most of their length, while spines were considered shorter and more sharply tapered. An R script, *Rgasilus*, was written to compare the found spine-seta formula to a database comprising the spine-seta formulae of all other known continental African ergasilids, outputting the species with the most similar formulae and the number of differences in the formulae. This script is freely available here: https://github.com/ferrevandenberg/Rgasilus.

### 2.3 Morphometrics

A proportion of copepod specimens (*n* = 34) was measured for their total length (medial, from cephalosome to furcal rami, excluding setae of furcal rami), cephalosome length (medial, from anterior to posterior end of cephalosome), and cephalosome width (widest part of the cephalosome, perpendicular to the total length) (37). The resulting data were compared between the lakes using Mann–Whitney *U*-tests (*stats* package), since the assumptions for parametric tests were violated (normality assessed using histograms, Q-Q plots, and Shapiro–Wilk test; homoscedasticity tested with F-test) in RStudio v4.2.2 (38). Additionally, Spearman correlation tests were performed to infer a correlation between the body size of the host and MI of the parasite, as well as between the body size of the parasite and MI of the parasite. All statistical tests were visualised with boxplots (geom_boxplot) and scatterplots (geom_point) using *ggplot2* package (39).

### 2.4 DNA extractions, PCR, and Sanger sequencing

DNA extractions were carried out in a UV cabinet (using sterile and UV-irradiated materials) to prevent contamination. A random subsample of 32 copepod specimens from *L. niloticus* (*n* = 20) and *L. longispinis* (*n* = 12) from Lake Turkana, and 18 copepods from *L. niloticus* originating from Lake Albert were processed. An overview of the executed molecular work can be found in Supplementary Table 1.

A buffer of TNES (Tris, NaCl, EDTA, SDS) (195 μL) (heated at 50 °C to redissolve precipitates) and proteinase *K* (5 μL of 20 mg/mL) was added to the specimens for a digestion of the soft tissues in the VWR Thermomixer (3 h, 800 rpm, 55 °C). The carapaces were removed with a sterile entomological needle and stored in 70% EtOH at 4 °C for morphological analysis (see 2.2 Morphological identification of parasitic copepods). To enhance the precipitation of the DNA, Invitrogen™ tYeast RNA (1.5 μL of 10 mg/mL) was added, along with NaCl (65 μL of 5 M) and 96% EtOH (290 μL). The extracts were then stored overnight at −20 °C. The samples were spun down in a cooled centrifuge (4 °C, 18000 rcf) for 15 min. The pellet was washed twice with 70% EtOH in a cooled centrifuge (4 °C, 18000 rcf) for 5 min. The supernatant was removed, and the residual ethanol was evaporated in the UV cabinet. The elution buffer (50 μL of 0.1X TE with 0.02% Tween™ 20 Surfact-Amps™ Detergent Solution) was added, and the extracts were resuspended overnight at 4 °C to increase the yield. The DNA extracts were stored at −20 °C.

The extracted DNA was amplified via Polymerase Chain Reaction (PCR) using the 28S-F (5′-ACA ACT GTG ATG CCC TTA-3′) and 28S-R (5′-TGG TCC GTG TTT CAA GAC-3′) primers for a partial 28S ribosomal DNA (28S rDNA) fragment (7). The 18S-F (5′-AAG GTG TGM CCT ATC AAC-3′) and 18S-R (5′-TTA CTT CCT CTAAAC GCT-3′) primers were used for the amplification of a partial 18S ribosomal DNA (18S rDNA) fragment (7). For the amplification of the 28S and 18S markers, 2.00 μL of extracted DNA was combined with 2.50 μL of PCR buffer (1×), 0.50 μL of dNTPs (0.2 mM), 0.10 μL of Q5^®^ High-Fidelity DNA Polymerase (0.5 μM), 1.25 μL of the forward and reverse primers, respectively (for the 18S or 28S genetic marker) (0.5 μM), and 17.40 μL of ddH_2_O, for a total reaction volume of 25.00 μL. The amplification reaction was performed under the following conditions: initial denaturation at 94 °C for 5 min; 35 cycles of 94 °C for 30 s, 54 °C for 1 min, and 72 °C for 1 min; and a final extension at 72 °C for 10 min, before being stored at −20 °C (5). For a separate set of samples, for which the previous protocol did not perform well, the fragments of 28S and 18S rDNA were amplified via PCR using MangoMix™. For each 2.00 μL of DNA extract, 12.50 μL of MangoMix™ (1×), 0.50 μL of MgCl_2_ (1 mM), 1.25 μL of the forward and reverse primers, respectively (the same primers as in the previous protocol) (0.5 μM), and 7.50 μL of ddH_2_O were added, for a total reaction volume of 25.00 μL. The amplification reaction consisted of an initial denaturation at 94 °C for 2 min; 39 cycles of 94 °C for 1 min, 50 °C for 1 min, and 72 °C for 1 min 30 s; and a final extension at 72 °C for 7 min, before being stored at −20 °C.

The generic primers LCO1490 and HCO2198 (40) targeting a fragment of mitochondrial cytochrome *c* oxidase subunit I (COI) mtDNA gene did not work for our samples. Therefore, in-house primers to amplify fragments of the mitochondrial cytochrome *c* oxidase subunit I (COI) gene were designed *de novo* through alignment of the mitogenomes of *E. kandti* (PQ276880.1) and a COI fragment of *Ergasilus mirabilis* Oldewage & van As 1987 (OR448770) using Primer3 v2.3.7 using default settings as implemented in Geneious Prime v2024.0.^1^ A 1317 bp fragment of the extracted DNA was amplified using primers 1,189-F (5′ - CCTGACATGGCTTTCCC - 3′) and 2,505-R (5′ - TCAAAGAGTTATGAGCCCTT - 3′).

For each 2.00 μL of DNA extract, 12.50 μL of MangoMix™ (1×), 0.50 μL of MgCl_2_ (1 mM), 1.25 μL of the forward and reverse primers, respectively (0.5 μM), and 7.50 μL of ddH_2_O were added, bringing the total to 25.00 μL per reaction. The amplification reaction was performed under the following conditions: initial denaturation at 94 °C for 2 min; 39 cycles of 94 °C for 1 min, 56 °C for 1 min, and 72 °C for 1 min 30 s; and a final extension at 72 °C for 7 min.

The success of the amplification reactions (18S rDNA, 28S rDNA, COI mtDNA) was verified using gel electrophoresis. The PCR products were purified using the Thermo Fisher Scientific™ GeneJET PCR Purification Kit following the protocols of the manufacturer. Sanger sequencing with the PCR primers was outsourced to Microsynth and Macrogen. The overview of the PCR and sequencing success is presented in Supplementary Table 1. The newly generated sequences were deposited in GenBank (Table 2).

**Table 2:** Copepod species (with haplotypes of *E. ereimia* sp. nov. from the concatenated 18S-28S rDNA phylogeny in Figure 9 indicated in brackets), specimen ID (with lake of origin indicated in brackets; LT for Lake Turkana, LA for Lake Albert), host species, GenBank accession numbers, and reference study of the sequences used in the phylogenetic analysis and generated by this study.

Copepod species	Copepod specimen ID	Host species	GenBank accession number			Reference study
			18S rDNA	28S rDNA	COI mtDNA	
*Acusicola margulisae* Santacruz, Morales-Serna, Leal-Cardín, Barluenga & de León 2020	824 N	–	MN852694	MN852851.1	–	Santacruz et al. (41)
*Dermoergasilus madagascarensis* Míč, Řehulková, Šimková, Razanabolana & Seifertová 2024	sp_1	*Paretroplus polyactis* Bleeker 1878	PP115568.1	PP115569.1	–	Míč et al. (3)
*Ergasilus anchoratus* (Markevich 1946)	EAH	*Tachysurus fulvidraco* (Richardson 1846)	DQ107564	DQ107528	–	Song et al. (7)
*Ergasilus arenalbus* Van der Spuy, Narciso, Hadfield, Wepener & Smit 2024	P1045	*Amblyrhynchote honckenii* (Bloch 1785)	PQ451954, PQ451956	PQ451957, PQ451958	–	Van der Spuy et al. (21)
*Ergasilus briani* Markevich 1933	EBN	*Misgurnus anguillicaudatus* (Cantor 1842)	DQ107572	DQ107532	–	Song et al. (7)
*Ergasilus caparti* Míč, Řehulková & Seifertová 2023	SPER_67	*Spathodus erythrodon* Boulenger 1900	OQ407469	OQ407474	–	Míč et al. (3)
*Ergasilus ereimia* sp. nov. (a)	Cop71 (LT)	*Lates longispinis* Worthington 1932	–	–	–	This study
*E. ereimia* sp. nov. (b)	Cop5 (LT)	*Lates niloticus* (Linnaeus 1758)	PX584523	PX584520	–	This study
	Cop6 (LT)	*L. niloticus*	PX584529	PX584521	PX513773	This study
	Cop9 (LT)	*L. niloticus*	PX584527	PX584511	PX513772	This study
*E. ereimia* sp. nov. (c)	Cop180 (LA)	*L. niloticus*	PX584526	PX584518	–	This study
*E. ereimia* sp. nov. (d)	Cop72 (LT)	*L. longispinis*	–	–	–	This study
*E. ereimia* sp. nov. (e)	Cop70 (LT)	*L. longispinis*	–	–	PX513778	This study
*E. ereimia* sp. nov. (f)	Cop10 (LT)	*L. niloticus*	PX584528	PX584510	PX513775	This study
	Cop11 (LT)	*L. niloticus*	PX584524	PX584512	PX513776	This study
	Cop185 (LA)	*L. niloticus*	PX584525	PX584515	–	This study
*E. ereimia* sp. nov. (g)	Cop192 (LA)	*L. niloticus*	–	–	–	This study
*E. ereimia* sp. nov. (h)	Cop15 (LT)	*L. niloticus*	–	–	PX513777	This study
*E. ereimia* sp. nov. (i)	Cop7 (LT)	*L. niloticus*	–	PX584522	PX513774	This study
	Cop181 (LA)	*L. niloticus*	–	PX584519	PX513780	This study
*E. ereimia* sp. nov. (j)	Cop12 (LT)	*L. niloticus*	–	–	–	This study
*E. ereimia* sp. nov. (k)	Cop193 (LA)	*L. niloticus*	–	–	–	This study
*E. ereimia* sp. nov. (l)	Cop179 (LA)	*L. niloticus*	–	PX584517	PX513779	This study
	Cop182 (LA)	*L. niloticus*	–	PX584513	–	This study
	Cop183 (LA)	*L. niloticus*	–	PX584514	PX513781	This study
	Cop187 (LA)	*L. niloticus*	–	PX584516	–	This study
*Ergasilus chintensis* Van der Spuy, Narciso, Hadfield, Wepener & Smit 2024	P1047	*A. honckenii*	PQ451955	PQ451959	–	Van der Spuy et al. (21)
*Ergasilus hypomesi* Yamaguti 1936	EHX	*Acanthogobius hasta* (Temminck & Schlegel 1845)	DQ107573	DQ107539	–	Song et al. (7)
*Ergasilus kandti* van Douwe 1912	5	*Tylochromis polylepis* (Boulenger 1900)	PQ249840	PQ249840	–	Jansen et al. (5)
*E. kandti*	6	*T. polylepis*	PQ249839	PQ249841	–	Jansen et al. (5)
*E. kandti*	11	*T. polylepis*	–	PQ249842	–	Jansen et al. (5)
*E. kandti*	13	*T. polylepis*	–	PQ249843	–	Jansen et al. (5)
*Ergasilus macrodactylus* (Sars 1909)	GNPE_159	*Gnathochromis permaxillaris* (David 1936)	OQ407465.1	OQ407470	–	Míč et al. (3)
*Ergasilus megacheir* (Sars 1909)	SIDI_EM2	*Simochromis diagramma* (Günther 1893)	OQ407466.1	OQ407471	–	Míč et al. (3)
*Ergasilus mirabilis* (Oldewage & van As 1987)	P38K-38VR	*Clarias gariepinus* Burchell 1822	OR449753.1	OR449755.1	–	Fikiye et al. (11)
*E. mirabilis*	P49K-72ZR	*C. gariepinus*	OR449754.1	OR449756.1	–	Fikiye et al. (11)
*Ergasilus parasarsi* Míč, Řehulková & Seifertová 2023	OPNA_99	*Ophthalmotilapia nasuta* (Poll and Matthes 1962)	OQ407467	OQ407473	–	Míč et al. (3)
*Ergasilus parasiluri* (Yamaguti 1936)	PPN	*Silurus asotus* Linnaeus 1758	DQ107567	DQ107537	–	Song et al. (7)
*E. parasiluri*	PPH	*Tachysurus fulvidraco* (Richardson 1846)	DQ107568	DQ107536	–	Song et al. (7)
*Ergasilus parvus* Míč, Řehulková & Seifertová 2023	SPER_70	*S. erythrodon*	OQ407468	OQ407472	–	Míč et al. (3)
*Ergasilus peregrinus* Heller 1865	EPG	*Siniperca chuatsi* (Basilewsky 1855)	DQ107577	DQ107531	–	Song et al. (7)
*Ergasilus scalaris* Markevich 1940	ESW	*Pseudobagrus vachellii* (Richardson 1846)	DQ107565	–	–	Song et al. (7)
*E. scalaris*	ESC	*Tachysurus dumerili* (Bleeker 1864)	DQ107566	DQ107538	–	Song et al. (7)
*Ergasilus sieboldi* von Nordmann 1832	U_Jezu_9Erg	*Perca fluviatilis* Linnaeus 1758	MW810238	MW810242	–	Kvach et al. (6)
*Ergasilus tumidus* Markevich 1933	ETP	*Acheilognathus taenianalis* (Günther 1873)	DQ107569	DQ107534	–	Song et al. (7)
*E. tumidus*	EXK	*A. taenianalis*	DQ107570	DQ107533	–	Song et al. (7)
*E. tumidus*	EXM	*A. taenianalis*	DQ107571	DQ107535	–	Song et al. (7)
*Ergasilus wilsoni* (Markevich 1933)	LEGOPOE014	Plankton sampling	KR048765.1	KR048843.1	–	Baek et al. (42)
*Ergasilus yaluzangbus* Kuang & Qian 1985	EYJ	*Oxygymnocypris stewartia* (Lloyd 1908)	DQ107578	DQ1075540	–	Song et al. (7)
*Ergasilus* sp.	LEGOPOE013	–	–	KR048842.1	–	Unpublished
*Lamproglena chinensis* Yü 1937	LCW	*Channa argus* (Cantor 1842)	DQ107553	DQ107545	–	Song et al. (7)
*Lamproglena clariae* Fryer 1956	UL241	*C. gariepinus*	OR242503.1	OR338196.1	–	Rindoria et al. (43)
*L. clariae*	UL242	*C. gariepinus*	OR242504.1	–	–	Rindoria et al. (43)
*Lamproglena cleopatra* Humes 1975	UL237	*Labeo victorianus* Boulenger 1901	OR242502.1	OR338170.1	–	Rindoria et al. (43)
*Lamproglena orientalis* Markevich 1936	LOQ	*Chanodichthys dabryi* Bleeker 1871	DQ107549	DQ107542	–	Song et al. (7)
*L. orientalis*	LOH	*Chanodichthys erythropterus* (Basilewsky 1855)	DQ107551	DQ107541	–	Song et al. (7)
*L. orientalis*	LOM	*Chanodichthys mongolicus* (Basilewsky 1855)	DQ107550	DQ107543	–	Song et al. (7)
*L. orientalis*	LOC	*Squaliobarbus curriculus* (Richardson 1846)	DQ107552	DQ107544	–	Song et al. (7)
*Lernaea cyprinacea* Linnaeus 1785	LCC	*C. erythropterus*	DQ107556	DQ107547	–	Song et al. (7)
*L. cyprinacea*	LCE	*Cyprinus carpio* Linnaeus 1758	DQ107555	–	–	Song et al. (7)
*L. cyprinacea*	LCH	*Hemiculter leucisculus* (Basilewsky 1855)	DQ107554	DQ107546	–	Song et al. (7)
*L. cyprinacea*	LCM	*Opsariichthys bidens* Günther 1873	DQ107557	DQ107548	–	Song et al. (7)
*Mytilicola intestinalis* Steuer 1902	Mi2	*Mytilus edulis* Linnaeus 1758	HM775187	–	–	Elsner et al. (44)
*M. intestinalis*	Mo6	*M. edulis*	HM775188	–	–	Elsner et al. (44)
*Mytilicola orientalis* Mori 1935	Mo10	*Magallana gigas* (Thunberg 1793)	HM775190	–	–	Elsner et al. (44)
*M. orientalis*	Mo9	*M. gigas*	HM775189	–	–	Elsner et al. (44)
*Neoergasilus japonicus* (Harada 1930)	Babice2_3Neoerg	*Lepomis gibbosus* (Linnaeus 1758)	MW810236	MW810240	–	Kvach et al. (6)
*N. japonicus*	Hvezda	*L. gibbosus*	MH167969	MH167967	–	Ondračková et al. (45)
*N. japonicus*	Rohlik	*L. gibbosus*	MH167970	MH167968	–	Ondračková et al. (45)
*N. japonicus*	LEGOPOE015	Plankton sampling	KR048752.1	KR048823.1	–	Baek et al. (42)
*Paraergasilus brevidigitus* Yin 1954	PBL	*C. carpio*	DQ107576	DQ107530	–	Song et al. (7)
*Paraergasilus longidigitus* (Yin 1954)	Pahrbek_10_11_12_13Paraerg	*Abramis brama* (Linnaeus 1758), *Perca fluviatilis* Linnaeus 1758, *Scardinius erythrophthalmus* (Linnaeus 1785)	MW810239	MW810243	–	Kvach et al. (6)
*Paraergasilus medius* Yin 1956	PMC	*Ctenopharyngodon Idella* (Cuvier & Valenciennes 1844)	DQ107574	DQ107529	–	Song et al. (7)
*P. medius*	PMQ	*Mylopharyngodon piceus* (Richardson 1846)	DQ107575	–	–	Song et al. (7)
*Pseudomyicola spinosus* (Raffaele & Monticelli 1885)	LEGOPOE025	Plankton sampling	KR048751	KR048822.1	–	Baek et al. (42)
*Sinergasilus major* (Markevich 1940)	SMC	*C. idella*	DQ107558	DQ107524	–	Song et al. (7)
*S. major*	SMG	*Elopichthys bambusa* (Richardson 1845)	DQ107560	–	–	Song et al. (7)
*S. major*	PE2PE8	*Silurus glanis* Linnaeus 1758	MZ047814	MZ047815	–	Dos Santos et al. (46)
*S. major*	SMCH	*Squaliobarbus curriculus* (Richardson 1846)	DQ107559	–	–	Song et al. (7)
*Sinergasilus polycolpus* (Markevich 1940)	SPL	*Hypophthalmichthys molitrix* (Valenciennes 1844)	DQ107563	DQ107525	–	Song et al. (7)
*Sinergasilus undulatus* (Markevich 1940)	SUJ	*Carassius auratus* (Linnaeus 1758)	DQ107562	DQ107527	–	Song et al. (7)
*S. undulatus*	SUL	*C. carpio*	DQ107561	DQ107526	–	Song et al. (7)
*Therodamas longicollum* Oliveira, Correa, Adriano & Tavares-Dias 2021	Jarilandia	*Leporinus fasciatus* (Bloch 1794)	MW652731	–	–	Oliveira et al. (47)

### 2.5 Genetic data analysis

A pairwise alignment per sample was performed for the obtained forward and reverse sequences of the partial 18S rDNA, 28S rDNA, and COI mtDNA genetic markers using the MUSCLE algorithm (48) with default settings in Geneious Prime v2024.0. The quality of the sequences was verified visually using the sequencing chromatograms and then they were trimmed accordingly. Published sequences of representatives of Ergasilidae were retrieved from GenBank, with the choice of outgroups according to Jansen et al. (5) (Table 2). The sequences were first aligned by marker with our newly generated sequences. Intra- and interspecific genetic distances for the fragments of the 18S rDNA, 28S rDNA, and COI mtDNA markers were calculated in MEGA 12.0 using the Kimura 2-parameter (K2P) model (49) allowing for transitions + transversions as substitutions, with gamma-distributed rates among sites (Gamma parameter: 4.00), and complete deletion of gaps/missing data (following settings as in Wu et al. (50)). The sequences of the fragments of the 18S and 28S rDNA genetic markers were concatenated (3, 6). Identical sequences were combined into unique haplotypes using FaBox v1.61 (51). IQTree v2.3.2 (52) was used to reconstruct the phylogenetic relationships using the Maximum Likelihood (ML) criterion. ModelFinder was employed to identify the best model with partition merging (53). To calculate the support values, the Ultra-Fast Bootstrap (UfBoot) approximation and the Shimodaira-Hasegawa (SH)-like approximate likelihood ratio test (SH-aLRT) were used (54), each with 10,000 replicates. The visualisation and editing of the ML tree were performed in FigTree v1.4.4, and RStudio v4.2.2, utilising the packages *car* (55), *reshape* (56)*, dplyr* (57), *ggplot2* (39), *ggrepel* (58), *ggraph* (59), *stringr* (60), *tibble* (61), *pbapply* (62), *phytools* (63), *treeio* (64), *ggtree* (64)*, Cairo* (65), and *tidy* (66). Finally, the labels were added to the figure in Affinity Designer v2.5.6. An additional alignment was conducted for the fragment of the COI mtDNA sequences of the samples from Lakes Turkana and Albert using the MUSCLE algorithm with default settings (Supplementary Table 1) to investigate the intraspecific relationships. An Integer Neighbour Joining Net haplotype network was constructed from this COI mtDNA alignment in Popart v1.7 (67).

## 3 Results

### 3.1 Parasitic infections on lates perches in lakes Turkana and Albert

A total of 1,645 specimens of parasitic copepods were retrieved from the gills of 15 host specimens (Table 1). In Lake Turkana, for *L. niloticus* (*n* = 4), a prevalence of 100% and a mean infection intensity of 164.5 copepods were observed. For *L. longispinis* (*n* = 6), the prevalence was also 100%, with a mean infection intensity of 156.2 copepods (Table 1). In Lake Albert, for *L. niloticus* (*n* = 5), a prevalence of 80% (with only one uninfected host) and a mean infection intensity of ten copepods were observed (Table 1).

Additionally, seven specimens of the monopisthocotylan flatworm identified as *Dolicirroplectanum lacustre* (Thurston & Paperna 1969)—based on the criteria described in Kmentová et al. (68) and Thys et al. (69)—were found on the screened hosts from Lake Turkana and were mounted on slides (Hasselt University collection; XXVI.3.25 – XXVI.3.30). For *L. niloticus* (*n* = 4) a prevalence of 25% was observed with a mean infection intensity of six flatworms, while for *L. longispinis* (*n* = 6) a prevalence of 17% and a mean infection intensity of one were observed. These specimens of *D. lacustre* were not analysed in detail in this study; see Supplementary Figure 1 for light microscopic pictures.

### 3.2 Morphological identification of copepods

**Figure 2:**
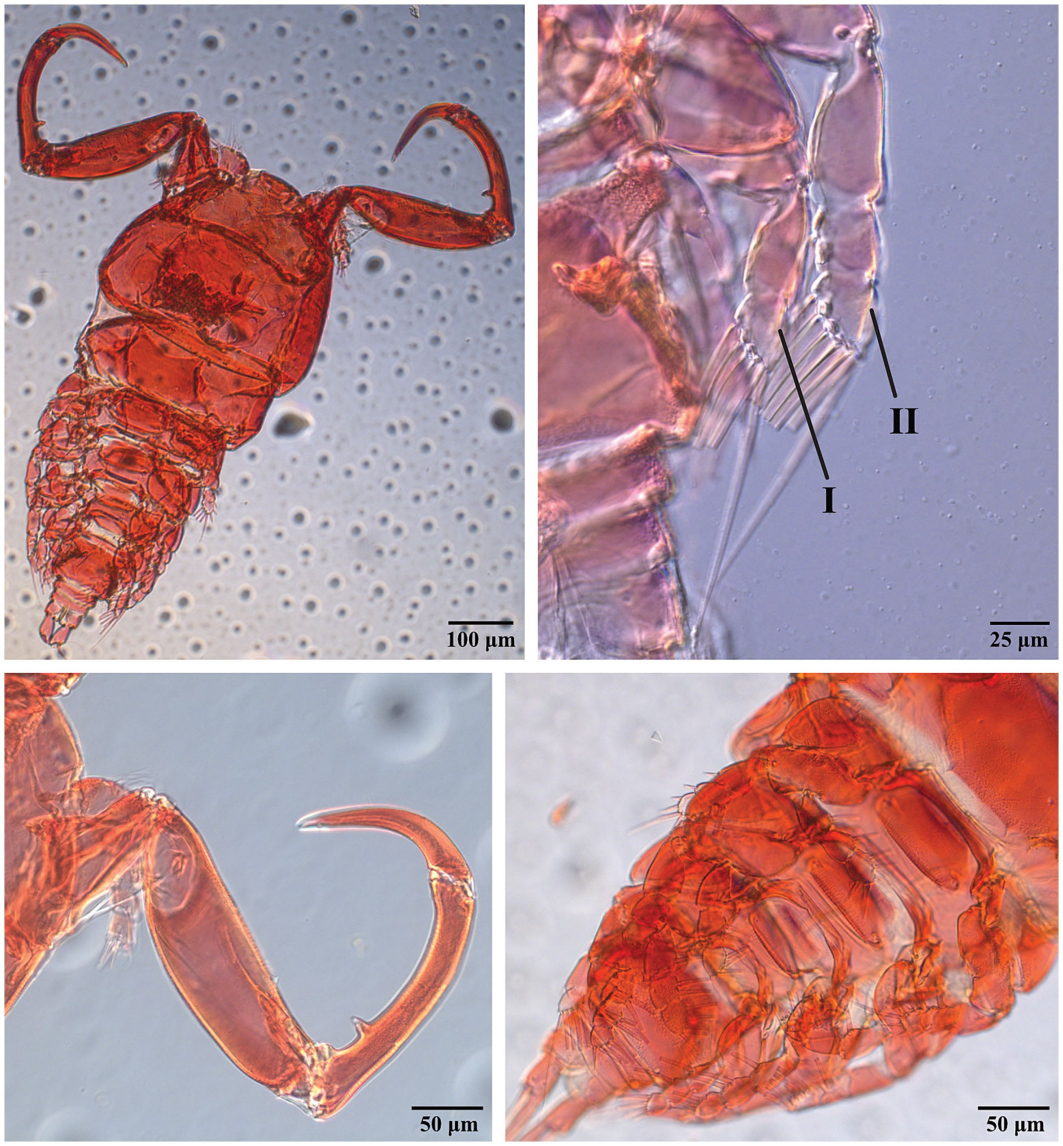
Light microscopic images of the full body **(A)** of digested *E. ereimia* sp. nov. specimen Cop11 (ex *L. niloticus*, Lake Turkana) at 100x; exopodites **(B)** of the fourth left swimming leg [**(B.I)**, two-segmented] and third left swimming leg [**(B.II)**, three-segmented] of digested specimen Cop15 (ex *L. niloticus*, Lake Turkana) at 400x; antenna **(C)** of digested specimen Cop11 (ex *L. niloticus*, Lake Turkana) at 200x; and metasome **(D)** of digested specimen Cop7 (ex *L. niloticus*, Lake Turkana) at 200x. The specimens were stained with Congo Red.

The copepod specimens mounted on microscopic slides were identified as belonging to *Ergasilus* (48 specimens of 48 mounted for light microscopy, 8 specimens of 22 mounted for confocal microscopy) based on the following combination of characteristics. The body is cyclopiform [Figure 2(A)], and the fourth swimming leg has a two-segmented exopodite [Figure 2(B.I)], whereas the first three swimming legs have a three-segmented exopodite [Figure 2(B.II)] (37, 70). Additionally, the first and second endopodal segments of the antenna are narrow and long, the latter of which is strongly curved and ornamented with sensilla supported by cuticular elevation, with at the terminal end of the antenna a single, curved, sharp claw that is smaller than the second endopod [Figure 2(C)] (71).

The spine-seta formula of the four pairs of biramous swimming legs and the rudimentary fifth leg is presented in Table 3 (36, 37), based on 16 specimens from Lake Turkana and 5 specimens from Lake Albert. Since no matching species was found based on morphology and spine-seta formula (see 3.3.4 Differential diagnosis), we propose the following description of a new species of *Ergasilus*.

**Table 3:** Spine-seta formulae of *E. ereimia* sp. nov. from Lakes Turkana and Albert of the five swimming legs, of *E. kandti* of the first four swimming legs compiled from Capart (23), and of *E. latus* of the first four swimming legs compiled from Fryer (27).

Species	Leg	Coxa	Basis	Exopodite	Endopodite
*E. ereimia* sp. nov.	**Leg 1**	0–0	0–0	I-0; I-1; II-5	0–1; 0–1; II-3
	**Leg 2**	0–0	I-0	I-0; 0–1; 0–6	0–1; 0–2; I-4
	**Leg 3**	0–0	0–0	I-0; 0–1; 0–6 [Figure 2(B.II)]	0–1; 0–2; I-4
	**Leg 4**	0–0	0–0	I-0; 0–5; − [Figure 2(B.I)]	0–1; 0–2; I-3
	**Leg 5**	–	–	I-0; I-1	–
*E. kandti*	**Leg 1**	/	/	I-0; **0–1**; II-5	0–1; 0–1; **II-4**
	**Leg 2**	/	/	I-0; 0–1; 0–6	0–1; 0–2; I-4
	**Leg 3**	/	/	**0–0**; 0–1; 0–6	0–1; 0–2; I-4
	**Leg 4**	/	/	**0–0**; 0–5; −	0–1; 0–2; I-3
*E. latus*	**Leg 1**	/	/	**0–0**; I-1; II-5	0–1; 0–1; **II-4**
	**Leg 2**	/	/	**0–0**; 0–1; 0–6	0–1; 0–2; I-4
	**Leg 3**	/	/	**0–0**; 0–1; 0–6	0–1; 0–2; I-4
	**Leg 4**	/	/	I-0; 0–5; −	0–1; 0–2; I-3

Roman symbols indicate number of spines, Arabic symbols indicate number of setae (36), slash (/) indicates undescribed structure, hyphen (−) indicates absent segment. Differences with the spine-seta formula of *E. ereimia* sp. nov. are marked in **bold**.

### 3.3 New species of *Ergasilus*

#### 3.3.1 Morphological description

Ergasilidae Burmeister 1835.

*Ergasilus* von Nordmann 1832.

*Ergasilus ereimia* sp. nov.

**Figure 3:**
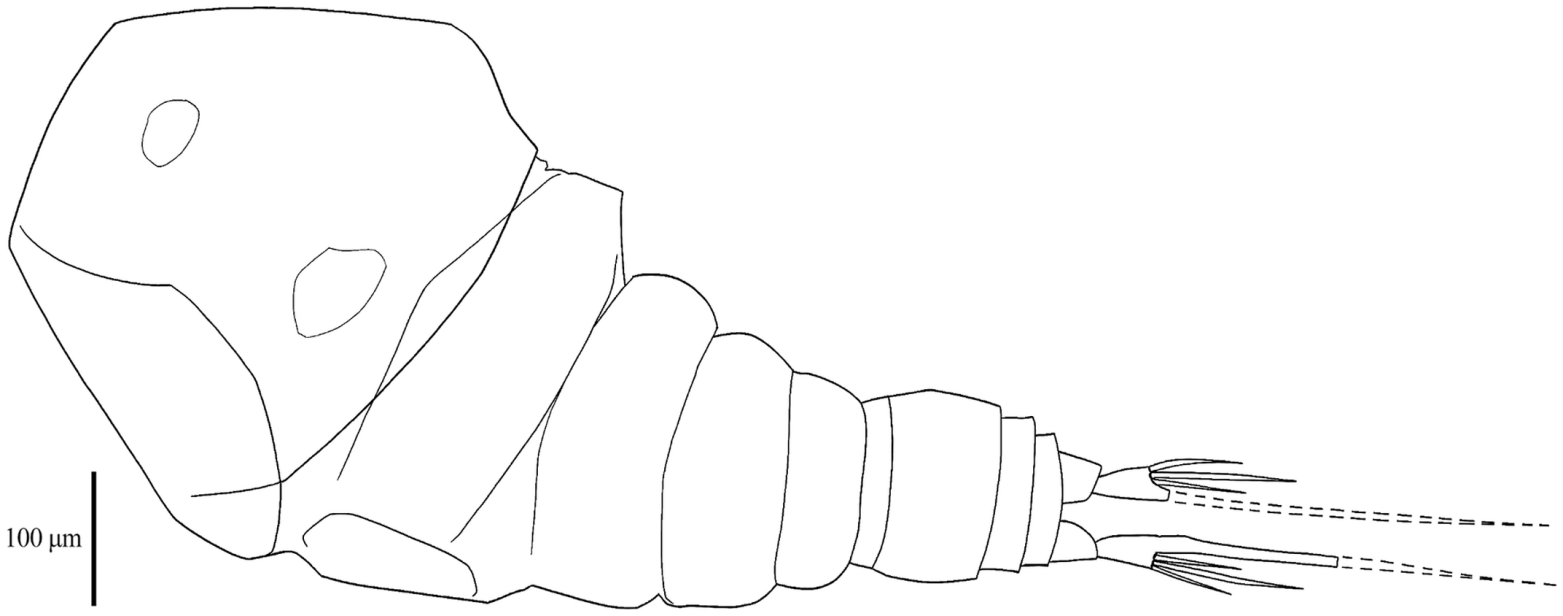
Drawing of the ventral body side of *E. ereimia* sp. nov. specimen Cop12 (ex *L. niloticus*, Lake Turkana). Dotted lines indicate missing structures (based on the opposite side of the same specimen, or on other specimens).

**Figure 4:**
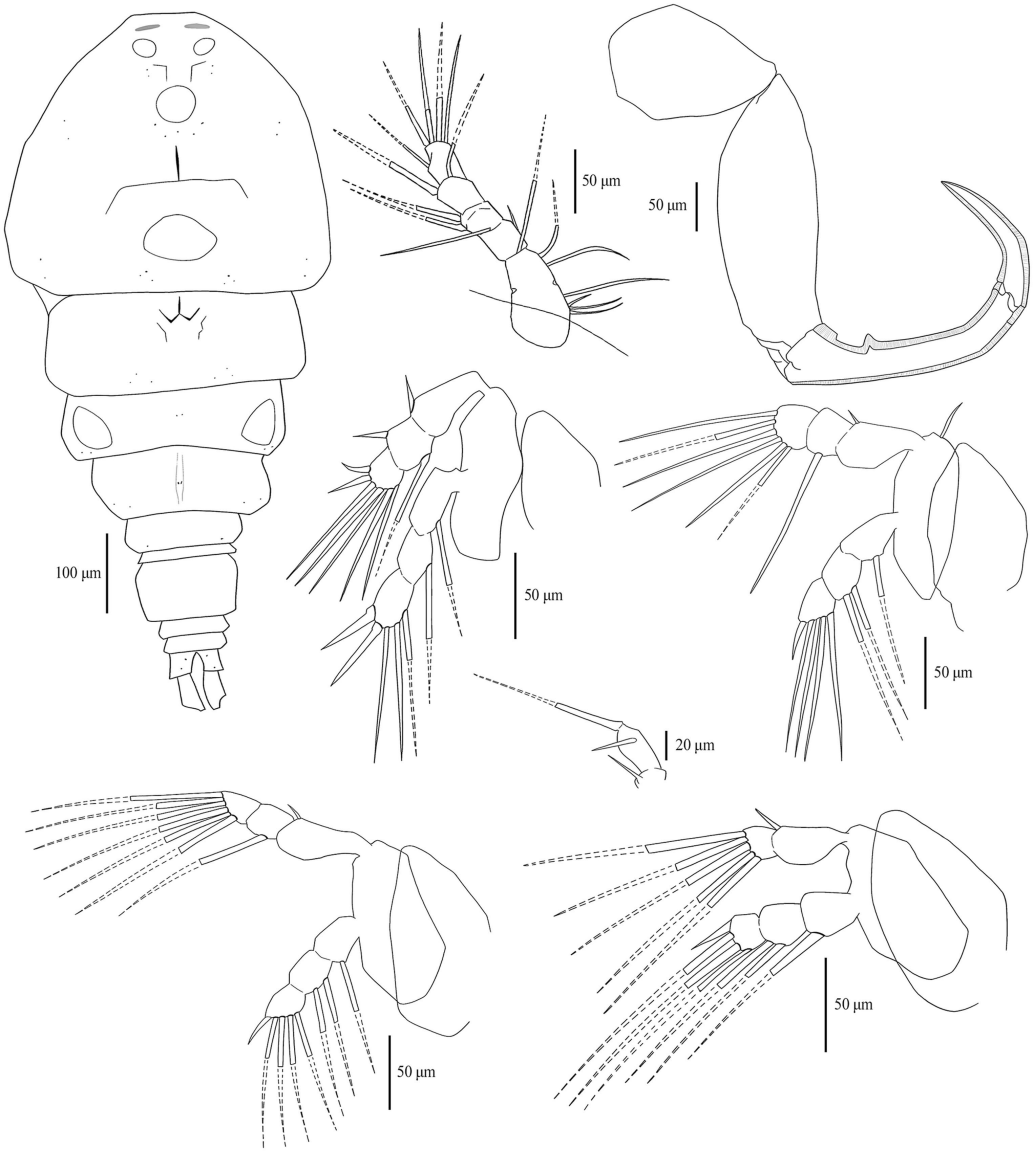
Drawings of the dorsal body side of (light microscopic) *E. ereimia* sp. nov. specimen Cop17 (ex *L. niloticus*, Lake Turkana), with ornamentation of (confocal microscopic) specimen Cop29 (ex *L. niloticus*, Lake Turkana) **(A)**; right antennule **(B)**; right antenna **(C)**; right leg 1 **(D)**; right leg 2 **(E)**; right leg 5 **(F)**; right leg 3 **(G)** and right leg 4 **(H)**. Dotted lines indicate missing structures (based on the opposite side of the same specimen, or on other specimens).

**Figure 5:**
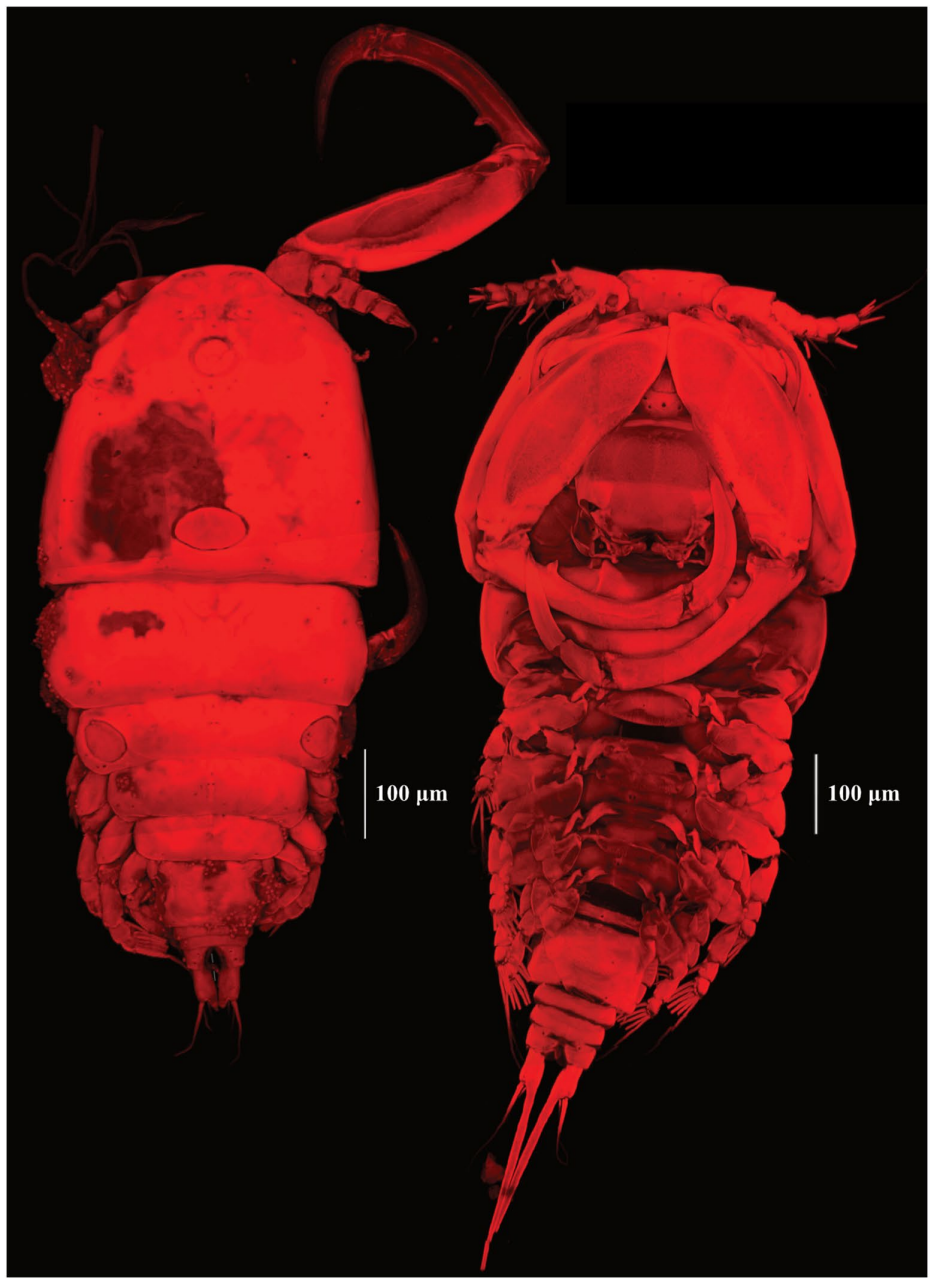
Confocal scans of undigested *E. ereimia* sp. nov. specimen Cop29 [dorsal habitus **(A)**] ex *L. niloticus*, Lake Turkana and specimen Cop70 [ventral habitus **(B)**], ex *L. longispinis*, Lake Turkana of this study. The specimens were stained with Congo Red.

**Figure 6:**
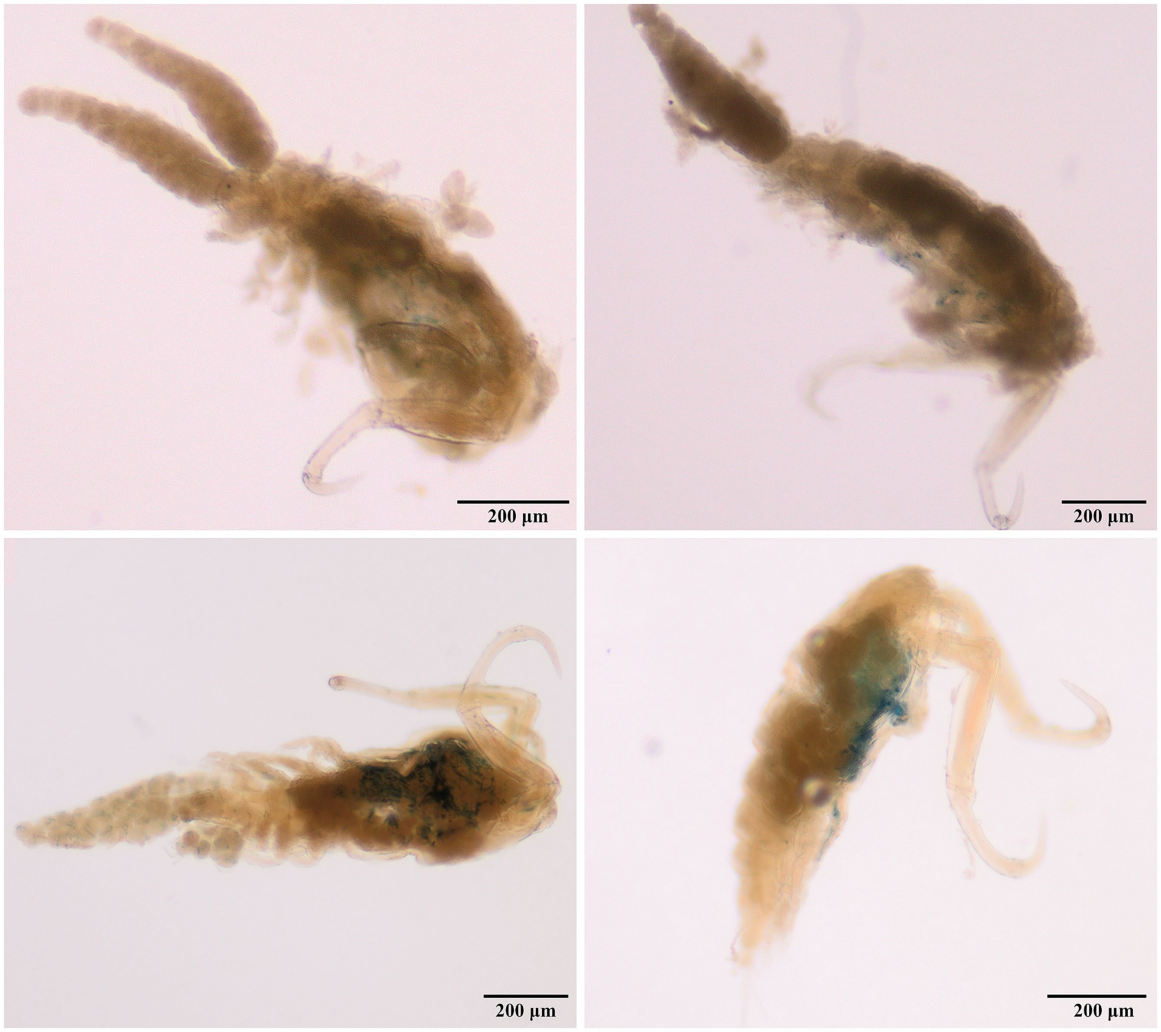
Light microscopic pictures (40x) of the undigested and unstained *E. ereimia* sp. nov. specimens Cop25 (ex *L. niloticus*, Lake Turkana) **(A)**, Cop27 (ex *L. niloticus*, Lake Turkana) **(B)**, Cop200 (ex *L. niloticus*, Lake Albert) **(C)**, and Cop201 (ex *L. niloticus*, Lake Albert) **(D)**. Specimens of Lake Albert **(C,D)** are more heavily pigmented.

Adult females were retrieved from gill filaments. Cephalosome wider than long (Table 4), broadest posteriorly. Trapezoid-shaped anterior, concave lateral sides. Clear segmentation between cephalosome and first thoracic segment [Figures 3, 4(A)]. Dorsal ornamentation of the cephalosome consists of an inverted T-structure medially, anterior to an ovoid structure, and posterior to a circular structure. Two smaller ovoid structures are anterior to the circular structure. Light anchor-shaped structure medial on the first thoracic segment. Two ovoid structures are laterally on the second thoracic segment [Figure 4(A)]. Antennules five-segmented. The first segment (counted from base upwards) is bigger with small lateral indents. Segments gradually decrease in width [Figure 4(B)]. Antennae four-segmented, long and slender. Second endopodal segment slightly arched with sensilla supported by a cuticular elevation on the posterior border, claw strongly curved, ending in a single point. Second endopod and claw with razor-like edges [hatched in Figure 4(C)]. Metasome five-segmented, gradually decreasing in length and width, bulged laterally with round edges. The fifth thoracic segment is covered by the fourth segment dorsally (Figure 5), but is still visible with light microscopy due to translucent carapace [Figures 3, 4(A)]. One pair of swimming legs per thoracic segment. Legs one to four are biramous [Figures 4(D,E,G,H)]. Exopodites and endopodites are three-segmented, except for the two-segmented exopodite of leg four [Figure 4(H)]. The fifth pair of swimming legs is two-segmented, with the first segment shorter and broader [Figure 4(F)], positioned dorsolaterally of the fifth thoracic segment (partly covered by the fourth thoracic segment). Intercoxal sclerites of the first four swimming legs are slender, unornamented, lateral ends directed posteriorly [Figures 2(A,D), 5(B)]. The interpodal plate of the first three swimming legs bulged posteriorly, with lateral indents creating a broad ridge-like structure, and lateral pores [Figures 2(D), 5(B)]. Interpodal plates of the fourth and fifth legs are missing. Urosome four-segmented (Figure 3). Genital segment wider than long, bulged laterally, wider than the fifth thoracic and first abdominal segment. The two abdominal segments and the anal segment gradually decrease in width. Anal segment split medially [Figures 3, 4(A)]. Furcal rami rectangular, longer than wide [Figures 3, 4(A)]. Four terminal setae on the posterior margin, with the innermost seta wider and longer. The central seta of the remaining three smaller setae is wider and longer than the other two (Figure 3). Body brownish-yellow with light to heavy cyan-blue pigmentation ventrally in ethanol-preserved specimens (mostly visible in unstained specimens, see Figure 6). Specimens from Lake Albert [Figures 6(C,D)] are more heavily pigmented than those from Lake Turkana [Figures 6(A,B)]. Specimens carry two egg sacs that can be as long as the whole body (Figure 6).

**Table 4:** The mean measurements and standard deviations of the total body length of the hosts (in mm) and *E. ereimia* sp. nov. (in μm), and the length and width of the cephalosome of *E. ereimia* sp. nov. from Lakes Turkana and Albert (in μm).

Water body	Host species	Host total length (mm)	*n* measured	*E. ereimia* sp. nov. total length (μm)	*E. ereimia* sp. nov. cephalosome length (μm)	*E. ereimia* sp. nov. cephalosome width (μm)
Lake Turkana	*L. niloticus*	332.85 ± 98.77	20	875.48 ± 40.78	343.75 ± 12.21	424.47 ± 34.10
	*L. longispinis*					
Lake Albert	*L. niloticus*	385.00 ± 7.81	14	827.22 ± 42.66	338.02 ± 22.69	391.18 ± 31.47

With *n*, the number of copepod specimens.

**Figure 7:**
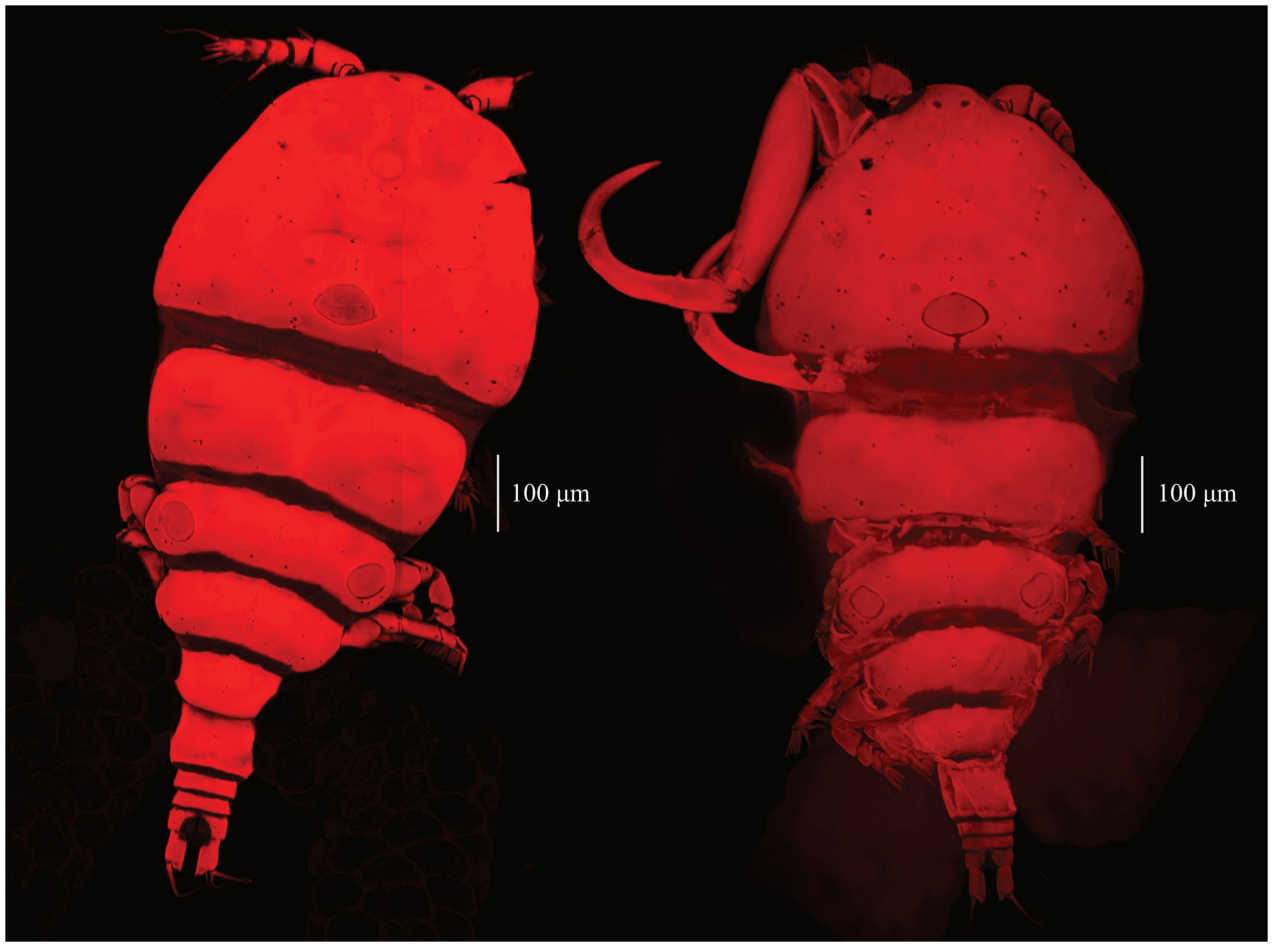
Confocal scans of the dorsal habitus of undigested *E. ereimia* sp. nov. specimens Cop93 **(A)** (ex *L. longispinis*, Lake Turkana) and Cop203 **(B)** (ex *L. niloticus*, Lake Albert). The specimens were stained with Congo Red.

The unique spine-seta formula of the swimming legs can be found in Table 3, and can also be observed in Figures 4, 5(B). Confocal scans were acquired of the dorsal and ventral habitus to visualise the ornamentation and the different body parts in a three-dimensional view (Figures 5, 7). Additional light microscopic images can be found in Figure 2 and Supplementary Figures 2, 3.

#### 3.3.2 Voucher material

The female specimens of *E. ereimia* sp. nov. were collected from Lakes Turkana and Albert (Figure 1), and were deposited in the collections of Hasselt University (UH; HU1098 - HU1113), and the Royal Museum for Central Africa (RMCA; RMCA_CRUST_58056 – RMCA_CRUST_58067).

Holotype: adult female HU1108.

Paratypes: adult females HU1098 - HU1107, HU1109 - HU1113.

#### 3.3.3 Etymology

The species epitheton of *E. ereimia* sp. nov. is derived from the ancient Greek word ἐρημία, which translates to desert. This refers to Lake Turkana, the type locality, which is one of the largest permanent desert lakes in the world.

#### 3.3.4 Morphological differential diagnosis

A morphological differential diagnosis was conducted with adult females of all 18 other known continental African species of *Ergasilus* (*E. kandti*, *E. latus, E. cunningtoni*, *E. flaccidus*, *E. inflatipes*, *E. lamellifer*, *E. macrodactylus*, *E. megacheir*, *E. mirabilis*, *E. nodosus*, *E. sarsi*, *E. briani*, *E. lizae*, *E. hypomesi*, *E. caparti*, *E. parasarsi*, *E. parvus*, and *E. ilani*) (Supplementary Table 2), revealing no correspondence with the descriptions, drawings, or spine-seta formulae provided by Kim (72), Schlebusch (37), Míč et al. (3), Fikiye et al. (11), and van der Spuy et al. (21). This differential diagnosis was conducted in accordance with Oldewage & van As (73), Míč et al. (3), and van der Spuy (21) on the basis of the cephalosome, the body and its segmentation, the antennae, the antennules, the genital somite, the furcal rami, the pigmentation, the ornamentation, the egg sacs, and the spine-seta formula of the swimming legs. For the spine-seta formula, the number of differences between the continental African species of *Ergasilus* and *E. ereimia* sp. nov. ranged from two (*E. hypomesi*) to 12 (*E. caparti* and *E. ilani*), with an average difference of seven segments (the respective differences are mentioned in Supplementary Table 2). The morphological differential diagnosis revealed *E. ereimia* sp. nov. to have a unique combination of morphological traits, as well as a unique spine-seta formula.

The only other ergasilids known to infect *L. niloticus* are *E. kandti* (22–26, 70) and *E. latus* (28), and the differences with these species are described below.

The specimens of *E. ereimia* sp. nov. from Lakes Turkana and Albert were compared with *E. kandti* based on the morphological description of van Douwe (22) (from Lake Albert) and Schlebusch (37), the morphological drawing and spine-seta formula of Capart (23) and Schlebusch (37), the confocal images from Jansen et al. (5), and voucher specimens of the UHasselt collection from Jansen et al. (5) (XXIII.1.41–50; and XXIII.2.01). The collection of the RMCA counts a single specimen of *E. kandti* (RMCA_CRUST_51547), which could not be located, and no type specimen was assigned in the description by van Douwe (22). The following differences in body shape and ornamentation were found. *E. ereimia* sp. nov. exhibits dorsal ovoid ornamentation posterior to the inverted T-structure on the cephalosome, while *E. kandti* does not; the metasome undergoes a less extreme reduction in the width of the thoracic segments compared to *E. kandti*, and the fifth leg is two-segmented (compared to a one-segmented fifth leg in *E. kandti*). Furthermore, the furcal rami of *E. ereimia* sp. nov. are rectangular in comparison to the square shape visible in *E. kandti*. *E. ereimia* sp. nov. differs in spine-seta formula for four segments from *E. kandti*. The following segments differ in spines and setae: the second segment of the exopodite of the first leg (I-1 for *E. ereimia* sp. nov.; 0–1 for *E. kandti*); the third segment of the endopodite of the first leg (II-3 for *E. ereimia* sp. nov.; II-4 for *E. kandti*); the first segment of the exopodite of the third leg (I-0 for *E. ereimia* sp. nov.; 0–0 for *E. kandti*); and the first segment of the exopodite of the fourth leg (I-0 for *E. ereimia* sp. nov.; 0–0 for *E. kandti*) (see Table 3, with differences marked in bold).

The specimens were also compared with *E. latus* based on the morphological descriptions and drawings by Fryer (27) and Schlebusch (37), and one specimen of the RMCA (RMCA_CRUST_51093) (no other specimens or types were available); the following differences were found. *E. ereimia* sp. nov. has a cephalothorax that is not fused to the first swimming leg as in *E. latus*, and antennules that are five-segmented, instead of six-segmented in *E. latus*. The antennae of *E. ereimia* sp. nov. are not as long and slender as in *E. latus*, with the second endopodal segment not swollen on the proximal end. The fifth swimming leg of *E. ereimia* sp. nov. is two-segmented, compared to only one-segmented in *E. latus.* The following four segments of the swimming legs differ in spines and setae: the first segment of the exopodite of the first leg (I-0 for *E. ereimia* sp. nov.; 0–0 for *E. latus*); the third segment of the endopodite of the first leg (II-3 for *E. ereimia* sp. nov.; II-4 for *E. latus*); the first segment of the exopodite of the second leg (I-0 for *E. ereimia* sp. nov.; 0–0 for *E. latus*); and the first segment of the exopodite of the third leg (I-0 for *E. ereimia* sp. nov.; 0–0 for *E. latus*) (see Table 3, with differences marked in bold).

#### 3.3.5 Remarks regarding morphology

Further morphological research is required to describe the mouthparts, the setation of the antennules, the third (small) endopod of the antenna, the ornamentation of the swimming legs (i.e., spinules and bristles), and the ornamentation of the urosomal segments. The description of these characteristics fell outside the possibilities of this study.

### 3.4 Morphometrics and infection parameters

**Figure 8:**
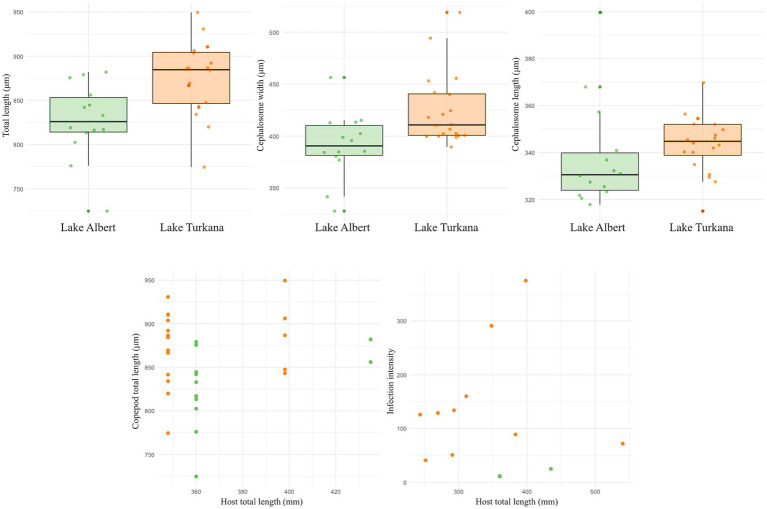
Boxplots comparing the total length **(A)**, cephalosome width **(B)**, and cephalosome length **(C)** of *E. ereimia* sp. nov. (in μm) between Lakes Albert (green) and Turkana (orange), as well as scatter plots of the relationship of the total host length (in mm) with the total length of *E. ereimia* sp. nov. (in μm) **(D)** and infection intensity **(E)**, respectively.

The total length of the body, as well as the length and width of the cephalosome, were measured for 20 copepod specimens of *E. ereimia* sp. nov. from Lake Turkana and 14 specimens of *E. ereimia* sp. nov. from Lake Albert (Table 4). Significant differences were found between specimens from the different lakes in the total body length (*p*-value = 0.001, Mann–Whitney *U*-test) [Figure 8(A)] and the width of the cephalosome (*p*-value = 0.006, Mann–Whitney *U*-test) [Figure 8(B)], both of which were larger in the Lake Turkana specimens (Table 4). No significant difference was found between the lakes in the length of the cephalosome (p-value = 0.066, Mann–Whitney *U*-test) [Figure 8(C)]. However, the cephalosomes of the specimens from Lake Turkana were slightly larger than those of Lake Albert (Table 4). We can conclude that the specimens of *E. ereimia* sp. nov. from Lake Turkana were larger overall than the specimens from Lake Albert.

The total length of *E. ereimia* sp. nov. showed no significant relationship with the total body length of the host (*p*-value = 0.4952, Spearman correlation test) [Figure 8(D)], nor was there a significant relationship between the total body length of the host and the infection intensity (p-value = 0.7003, Spearman correlation test) [Figure 8(E)].

### 3.5 Phylogenetic reconstruction

**Figure 9:**
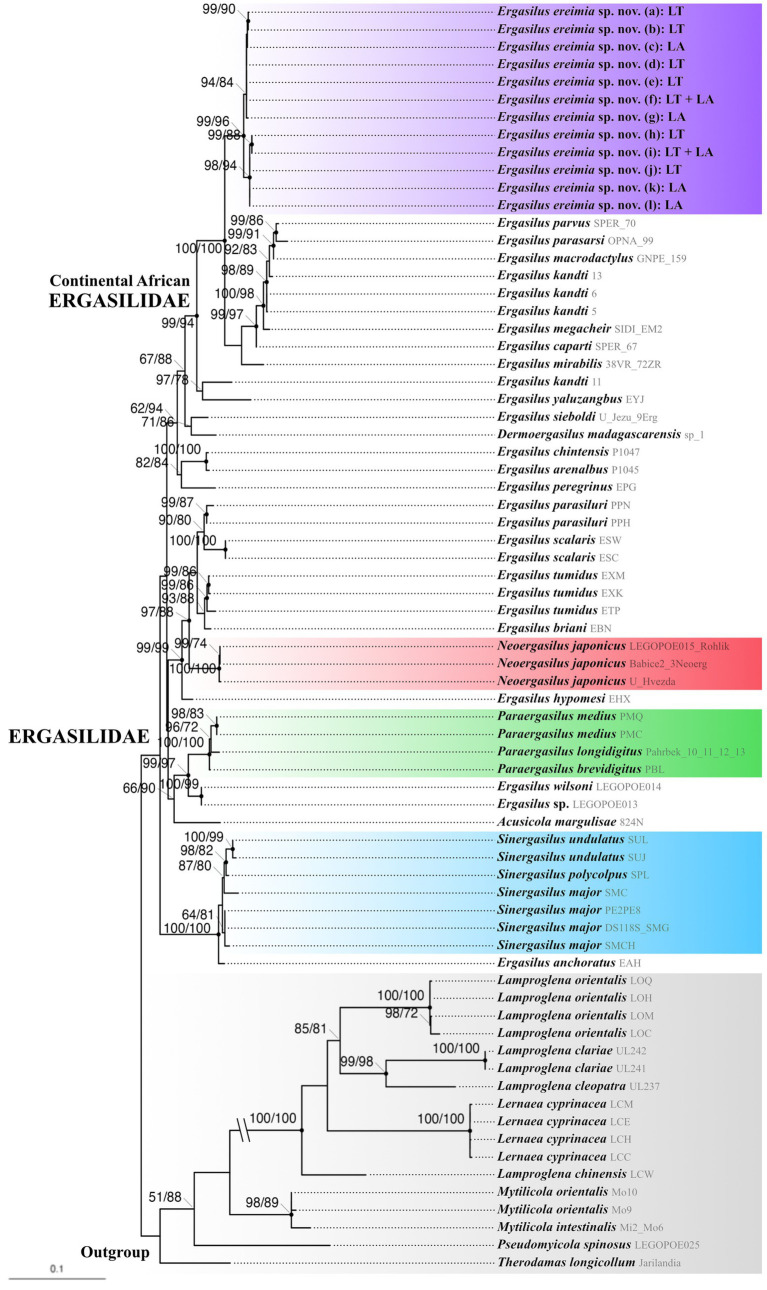
Phylogenetic tree of the concatenated alignment (18S–28S rDNA gene fragments) with UfBoot and SH-aLRT support values. A dot on a node indicates a highly supported clade (UfBoot ≥ 95 and/or SH-aLRT ≥80). The haplotypes are indicated in brackets (Table 2). The specimen IDs are specified by light grey text. The sequences of *Ergasilus ereimia* sp. nov. from this study are indicated in purple, with LT representing Lake Turkana and LA Lake Albert. *Neoergasilus* is indicated in red, *Paraergasilus* in green, and *Sinergasilus* in blue. The outgroup is indicated in grey. The scale bar indicates a branch length corresponding to 0.1 substitutions.

The ML phylogenetic tree based on the concatenated alignment (1,430 bp) of the partial 18S and 28S rDNA genetic markers can be found in Figure 9. The sequences of *E. ereimia* sp. nov. from Lakes Turkana and Albert form a distinct clade, with a sister clade containing the remaining continental African species of *Ergasilus* (*E. parvus*, *E. parasarsi*, *E. macrodactylus*, *E. kandti*, *E. megacheir*, *E. caparti*, and *E. mirabilis*). However, the continental African species of *Ergasilus* are rendered paraphyletic by the clade containing *E. yaluzangbus* (which only occurs in China; (7, 21)) and one of the sequences of *E. kandti* (PQ249842.1). The genus *Ergasilus* is rendered paraphyletic by *Dermoergasilus madagascarensis*, *Neoergasilus* Yin 1956 (*N. japonicus*), *Paraergasilus* Markevich 1937 (comprising *P. medius*, *P. longidigitus*, and *P. brevidigitus*), *Acusicola margulisae*, and *Sinergasilus* Yin 1949 (*S. undulatus*, *S. polycolpus*, *S. major*). Noteworthy, the sequences of *E. ereimia* sp. nov. from Lakes Turkana and Albert do not form distinct lineages based on 18S and 28S rDNA. *Paraergasilus* and *Sinergasilus* are found to be monophyletic.

### 3.6 Haplotype network and genetic distances

**Figure 10:**
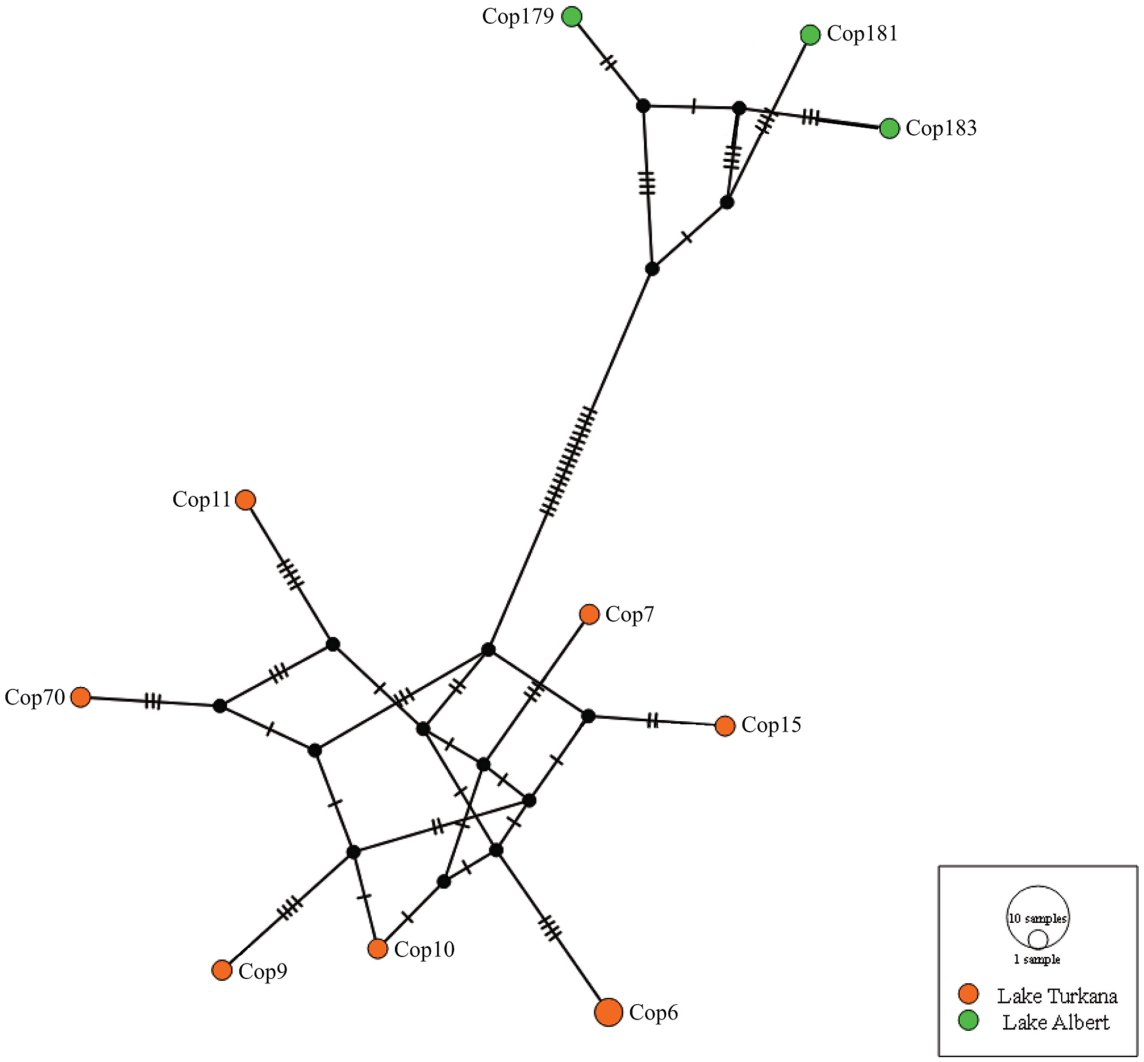
Haplotype network of the fragment of COI mtDNA genetic marker (1,122 bp) from *Ergasilus ereimia* sp. nov. of Lake Turkana (orange) and Lake Albert (green). All specimens were sampled from *L. niloticus*, except for Cop70, which was sampled from *L. longispinis*.

The haplotype network of *E. ereimia* sp. nov. based on a fragment of the COI mtDNA is presented in Figure 10. The sequences (alignment of 1,013 bp) from Lake Turkana (in orange) form a distinct group (separated by at least 18 mutations) from the sequences from Lake Albert (in green). For specimens from Lake Turkana, K2P distances for the COI mtDNA ranged from 0.004 to 0.014 (*n* = 7), while those from Lake Albert ranged from 0.006 to 0.011 (*n* = 3). In contrast, interlacustrine genetic distances were notably higher, ranging from 0.025 to 0.035.

The intra- and interspecific K2P genetic distances of all available sequences of members of Ergasilidae, calculated based on the fragments of the 18S rDNA, 28S rDNA, and the COI mtDNA genetic sequences, can be found in Table 5. The average interspecific distance over the fragment of 18S rDNA is 31 times greater than its intraspecific K2P distance (0.001 for intraspecific; 0.031 for interspecific), while the average interspecific K2P distance over the fragment of 28S rDNA is 154 times greater than its intraspecific distance (0.001 for intraspecific; 0.154 for interspecific).

**Table 5:** Genetic distance of intra- and interspecific comparisons for *E. ereimia* sp. nov. based on fragments of the 18S rDNA (796 bp), 28S rDNA (634 bp), and COI mtDNA (1,122 bp) sequences.

K2P distance	18S rDNA			28S rDNA			COI mtDNA		
	Min	Max	Average	Min	Max	Average	Min	Max	Average
Intraspecific	0.000	0.003	0.001	0.000	0.002	0.001	0.004	0.035	0.019
Interspecific	0.005	0.089	0.031	0.050	0.467	0.154	–	–	–

Interspecific distances were based on alignment with the following species: *E. ereimia* sp. nov.*, E. parvus*, *E. parasarsi*, *E. macrodactylus*, *E. kandti*, *E. megacheir*, *E. caparti*, *E. mirabilis*, *E. yaluzangbus*, *E. sieboldi*, *Dermoergasilus madagascarensis*, *E. chintensis*, *E. arenalbus*, *E. peregrinus*, *E. parasiluri*, *E. scalaris*, *E. tumidus*, *E. briani*, *Neoergasilus japonicus*, *E. hypomesi*, *Paraergasilus medius*, *P. longidigitus*, *P. brevidigitus*, *E. wilsoni*, *Acusicola margulisae*, *Sinergasilus undulatus*, *S. polycolpus*, *S. major*, and *E. anchoratus*.

## 4 Discussion

The morphological characterisation of *Ergasilus ereimia* sp. nov. from Lakes Turkana and Albert was conducted in accordance with the study of Oldewage & van As (73), Míč et al. (3), and van der Spuy (21). The findings demonstrated a sufficient amount of distinct characters, both in morphology and spine-seta formula, compared to the 18 other continental African representatives of Ergasilidae (Supplementary Table 2), including *E. kandti* and *E. latus* (see 3.3.4 Morphological differential diagnosis), which constitute the only previously reported copepod species infecting *L. niloticus* (22–26, 28, 70). Notably, interlacustrine variation in *E. ereimia* sp. nov. was limited to continuous morphological characters, such as coloration and body size, while no differences were observed in discrete diagnostic traits. The phylogenetic tree of the concatenated alignment of the partial 18S and 28S rDNA genetic markers (Figure 9) further showed that *E. ereimia* sp. nov. forms a distinct lineage. The partial 18S rDNA genetic marker evolves slowly and is therefore highly conserved among copepods (74), rendering it informative for resolving relationships at the family and genus levels, but not at the species level (75). The fragment of the 28S rDNA genetic marker provides the phylogenetic signal required to resolve potential relationships among species and genera (76, 77). The concept of DNA barcoding is predicated on the existence of a barcoding gap, whereby the interspecific genetic distances should exceed at least tenfold the intraspecific genetic distances as a commonly accepted criterion for delineating species (78). The average interspecific K2P distance (of copepod sequences included in this study) in the fragment of the 18S rDNA sequence is 31 times greater than the intraspecific distance (Table 5), while for the fragment of 28S rDNA, which has a higher resolution to delineate species, the average interspecific K2P distance is 154 times greater than the intraspecific distance (Table 5). Wu et al. (50) reported that for 110 sequences of the 28S rDNA genetic marker of planktonic calanoid species, the average interspecific distance was approximately 93 times greater, which is greatly exceeded by the barcoding gap observed in our findings. This suggests that *E. ereimia* sp. nov. cannot only be proposed as a novel species based on its morphology, but also on the basis of its distinct genetics. The fragment of the COI mtDNA genetic marker exhibits high mutation rates, enabling high-resolution species-level identification and the effective detection of intraspecific variation (79). Baek et al. (42) reported an average intraspecific K2P distance of 0.043 for the COI mtDNA genetic marker of 133 sequences of copepod species, while our fragment of the COI mtDNA sequences displayed an average intraspecific distance of 0.019 (Table 5), indicating that the genetic diversity within *E. ereimia* sp. nov. is comparatively low. However, the key evidence supporting the conclusion that the specimens from both lakes belong to the same species lies in the lack of divergence in the ribosomal markers (18S and 28S rDNA), which are more conserved and reliable for assessing species boundaries in copepods (76, 77). The consistency in these markers between populations originated from Lakes Albert and Turkana, respectively, confirms that *E. ereimia* sp. nov. constitutes a single, genetically cohesive species.

The advantage of the haplotype network is that it is model-free, thereby obviating the necessity to make assumptions about unknown evolutionary rates. This, however, also represents the largest limitation of this method. Although it is effective at visualising genetic diversity, its simplicity may not accurately reflect biological reality (67). The acquired haplotype network of the fragment of COI mtDNA (Figure 10) with its two haplogroups clearly shows that the populations from the lakes have genetically diverged from each other, with the interlacustrine K2P distances ranging from 0.025 to 0.035. In addition, morphological intraspecific variation was observed. For all measurements taken, the specimens of *E. ereimia* sp. nov. from Lake Turkana are larger, with the body length and cephalosome width being significantly larger (Table 4). The specimens of *E. ereimia* sp. nov. from Lake Albert are also more heavily pigmented than those from Lake Turkana (Figure 6). However, the absence of differences in discrete morphological characteristics between the lakes, combined with the low interlacustrine K2P distance of the fragment of the COI mtDNA (42, 80) and the low intraspecific K2P distances observed in the 18S rDNA, 28S rDNA and COI mtDNA markers (Table 5), support the conclusion that the populations from both Lake Albert and Lake Turkana belong to a single species. Such findings echo patterns observed in other freshwater parasites, notably the monopisthocotylan *D. lacustre* (also infecting lates perches), which displays substantial inter- and intralacustrine variation—including the presence of distinct morphotypes (with variation in continuous characteristics) and mitonuclear discordance between these morphotypes—while still being treated as a single species (68, 69). In contrast to *D. lacustre*, however, the morphological variation in *E. ereimia* sp. nov. remains minor, continuous, and inconsistent, without evidence for discrete morphotypes or reproductive isolation. Continuous intraspecific morphological variation has previously been observed in free-living copepods and can be influenced by environmental conditions, as demonstrated by Leinaas et al. (81) for the body size of calanoid copepods in Arctic and temperate waters. The different environmental conditions (or seasonal conditions at the time of sampling) of the lakes could be a possible explanation for the significant difference in body size of *E. ereimia* sp. nov. or for the difference in pigmentation. Sometimes subtle differences in discrete characteristics, such as the setation on the swimming legs, can be found when comparing with older descriptions of the same species, but this was attributed by Boxshall (82) to possible damage to the older specimens or to overlooking the characters.

We hypothesised that *E. ereimia* sp. nov. would be part of the clade containing all other continental African ergasilids. This proved to be correct, and *E. ereimia* sp. nov. forms a separate, well-supported clade sister to all other members of the continental African ergasilids (Figure 9). With the exception of one sequence (*E. kandti*; PQ249842.1), all other continental African ergasilids form a monophyletic clade, as was hypothesised by Song et al. (7) and Jansen et al. (5). However, in our phylogenetic reconstruction, the aberrant sequence of *E. kandti* from Zambia and the Tibetan *E. yaluzangbus* resolved as a sister clade to all other continental African species. It has previously been shown that *Ergasilus* is polyphyletic (3, 5–7). Our phylogenetic reconstruction further supports this non-monophyly of the genus, as it contains clades of the other genera *Neoergasilus*, *Paraergasilus,* and *Sinergasilus*. It is presumed that *Sinergasilus* is monophyletic (5, 7, 46). In Song et al. (7) and Kvach et al. (6), this genus was nested within *Ergasilus*, with *E. anchoratus* as a sister species of *Sinergasilus* (6, 46). These findings are further supported by the phylogenetic analysis in the present study. As was observed by Kvach et al. (6), the species of *Paraergasilus* (*P. medius*, *P. longidigitus* and *P. brevidigitus*) constitute the sister group of *Ergasilus wilsoni* in the constructed phylogeny (Figure 9, green), and *Ergasilus parasiluri* (Yamaguti 1936) (formerly named *Pseudergasilus parasiluri*) stands as the sister species of *Ergasilus scalaris* (Figure 9). The Malagasi species *Dermoergasilus madagascarensis* constitutes the sister species of the cosmopolitan *E. sieboldi* in our phylogenetic tree (Figure 9), as was previously also shown by Míč et al. (3). The phylogenetic analyses conducted in this study and their study did not reveal a close relationship between *D. madagascarensis* and the continental African ergasilids, which could indicate that this species did not originate in Africa (3). However, it is challenging to draw a definitive conclusion based on the limited amount of data available concerning African ergasilids.

Given the close association between parasites and their hosts, it is beneficial for the fishery sector to know the parasite–host (and parasite–parasite) interactions of novel species, since they could induce substantial host mortality (32). No significant correlation was found between the total body length of the host and the infection intensity [Table 1; Figure 8(E)]. Henriksen et al. (83) showed that fish body size is a predictor of the infrapopulation size in parasitic copepods, as is commonly reported in the literature (84, 85). However, lower levels of infection intensity have also been observed with increasing fish body size (85, 86). In this study, the lack of correlation between the fish body size and the infection intensity is presumably due to the non-normally distributed measurements of the total body length of the hosts. A specific size range of host specimens was procured from local merchants at the lakes to optimise the possible infection by monopisthocotylan parasites in the framework of ongoing research. The measurements of our host specimens (Table 4) do not represent the natural range of host body size in the lakes.

The lake of origin does influence the infection intensity of copepods based on the infection parameters (Table 1). Hosts from Lake Turkana have a remarkably higher intensity of infection than the hosts from Lake Albert. However, the sampling of host specimens at the lakes occurred at different months in different years (September 2022 in Lake Turkana; April 2019 in Lake Albert), as well as in different zones of the lake (shallow bay for Lake Turkana, open water for Lake Albert). It is also worthy of note that only the right gills of the hosts from Lake Albert were procured for screening for ectoparasites. Therefore, these infection parameters may not be an accurate representation of the natural host–parasite and parasite–parasite dynamics. For future studies, we recommend a thorough sampling throughout the lakes at different time periods, and potentially also other water bodies. Notably, the hosts from Lake Turkana were also much more heavily infected with the monopisthocotylan *D. lacustre* than the hosts from Lake Albert (7 specimens for Lake Turkana, 0 for Lake Albert). Gobbin et al. (87) observed a similar synergistic interaction between ectoparasitic copepods (*Lamproglena monodi* Capart 1944 and *Ergasilus lamellifer* Fryer 1961) and monopisthocotylan flatworms (*Cichlidogyrus* spp.) on haplochromine cichlid hosts from Lake Victoria. Another possible explanation could be that our host specimens were sampled in different periods. Since the life cycles of parasitic copepods are influenced by seasonality, the infection parameters could vary throughout the year (1). The different environmental conditions present in the lakes of origin may also exert an influence on the observed patterns of infection and morphology, since Lake Turkana is defined as an endorheic lake—a closed system—in contrast to Lake Albert, which is an exorheic lake that is connected to the Victoria Nile (15).

Alston et al. (88) hypothesised that the bright pigmentation of certain ergasilids might function as a tactic for the female copepods to bait the fish host to ingest them, after which it is thought the copepods prevent themselves from being swallowed by attaching their claw-like antennae to the fish’s gill rakers, to then move to the gills (89). Additionally, Byron (90) proved in laboratory and field experiments that vertebrate predators exhibit a preference for pigmented calanoid copepods (Ergasilidae belongs to Cyclopoida) through visual selection. Given that the water of Lake Albert is considerably more turbid than that of Lake Turkana (Secchi disk depth of 2–6 m for Lake Albert; 1–13 m for Lake Turkana) (91), the heavy pigmentation of the specimens of *E. ereimia* sp. nov. from Lake Albert may be a way to increase the chances of transmission to their host in the more turbid water. Unfortunately, we do not have the data to causally link these factors to the degree of pigmentation observed in our specimens. Further investigation into the influences of the host and the lake of origin could be conducted through comprehensive sampling, encompassing a range of hosts across diverse water bodies, and/or through the utilisation of experimental set-ups to assess potential correlations between the physico-chemical characteristics of the water, the host’s behaviour, and the parasites’ infection parameters.

## Data Availability

The datasets presented in this study can be found in online repositories. The names of the repository/repositories and accession number(s) can be found in the article/Supplementary material.

## Footnotes

1 https://www.geneious.com
