# Supplementary material for: A new ergasilid copepod from lates perches in East Africa: morphology, phylogenetics, and genetic structure of Ergasilus ereimia sp. nov
Source: Front Vet Sci. 2026 Feb 2;12:1699263. doi: 10.3389/fvets.2025.1699263 (PMC12908592; doi:10.3389/fvets.2025.1699263)
Supplement: Supplementary file 1 [file Data_Sheet_1.pdf]

Supplementary Material

1 Supplementary Figures and Tables

This document contains 3 supplementary figures and 2 supplementary tables.

1.1 Supplementary Figures

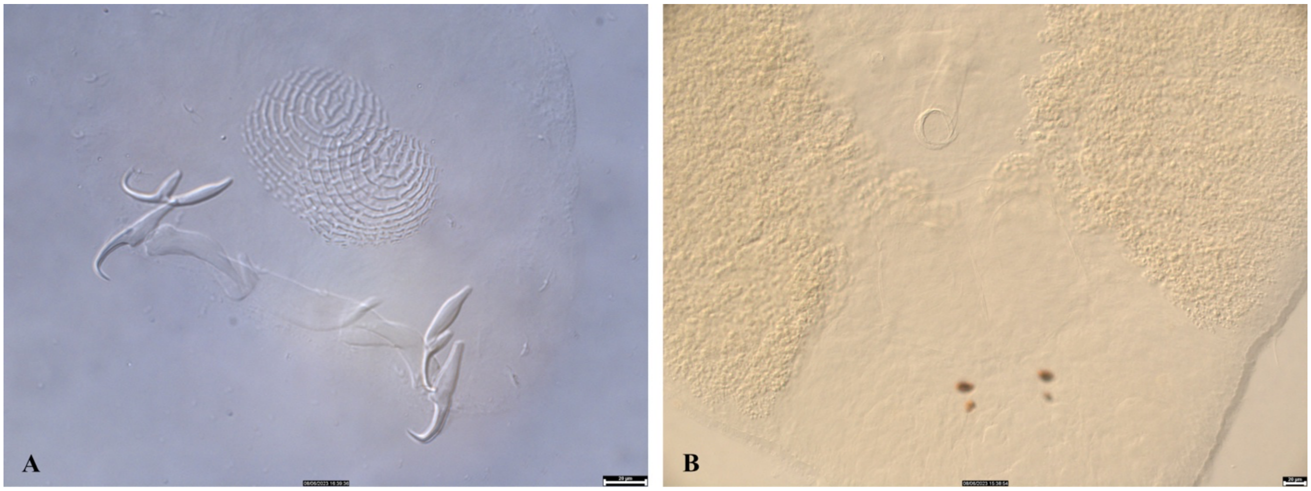

Supplementary Figure 1. Light microscopic pictures (400x) of the haptor (A) of *Dolicirroplectanum lacustre* specimen LL1-1 (ex *L. longispinis*, Lake Turkana), and of the male copulatory organ and eyespots (B) of LL1-4 (ex *L. longispinis*, Lake Turkana).

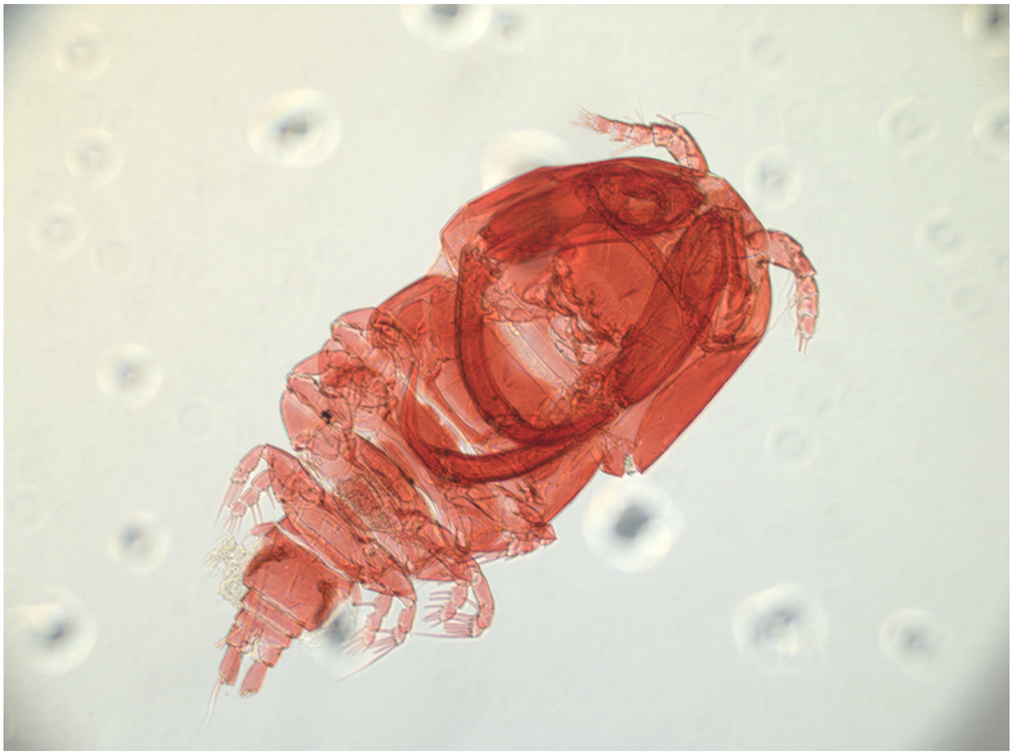

Supplementary Figure 2. Light microscopic picture (100x) of digested *Ergasilus ereimia* sp. nov. specimen Cop17 (ex *L. niloticus*, Lake Turkana). The specimen was stained with Congo Red.

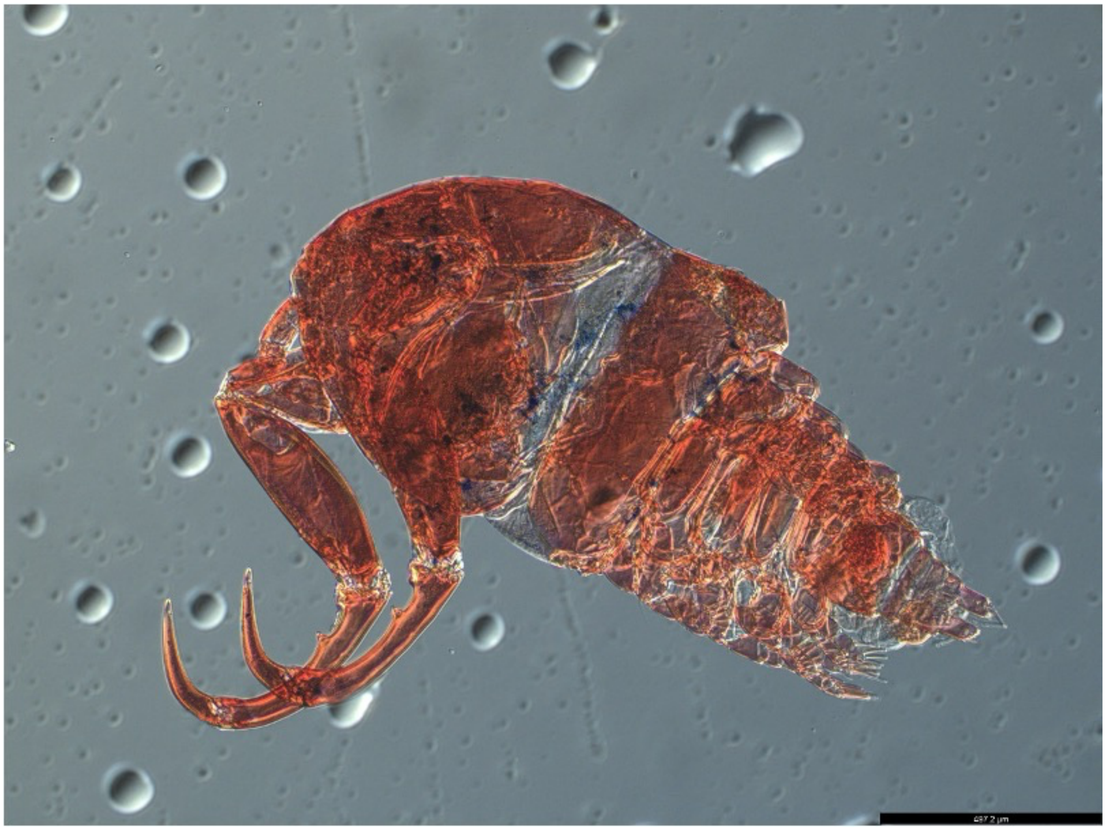

Supplementary Figure 3. Light microscopic picture (100x) of digested *Ergasilus ereimia* sp. nov. specimen Cop181 (ex *L. niloticus*, Lake Albert). The specimen was stained with Congo Red.

1.2 Supplementary Tables

Supplementary Table 1. Overview of the molecular work with the number of DNA extractions, PCR success and PCR reactions, and sequencing success, for the portions of the 28S rDNA, 18S rDNA, and COI mtDNA genetic markers for Lakes Turkana and Albert using the standard and MangoMix™ protocols (see 2.4 DNA extractions, PCR, and Sanger sequencing).

**Waterbody**	**Host species**	**DNA extractions**	**Protocol**	**PCR success / reactions**			**Sequencing success**		
				**28S**	**18S**	**COI**	**28S**	**18S**	**COI**
	L. niloticus,	32	Standard	7/22	6/22	0/6	11	8	7

Lake Turkana	*L. longispinis*		MangoMix	5/5	5/5	17/22			
Lake Albert	*L. niloticus*	18	Standard	9/22	3/22	-	8	3	3
			MangoMix	2/2	2/2	3/5			

Supplementary Table 2. Morphological comparison of *E. ereimia* sp. nov. with continental African species of *Ergasilus*.

***Ergasilus* species**	**Differences with *E. ereimia* sp. nov.**
E. cunningtoni	Antennules of *E. ereimia* sp. nov. five-segmented, compared to six in *E. cunningtoni*. Second endopodal segment of antennae of *E. ereimia* sp. nov. not indented anteriorly as in *E. cunningtoni*. The spine seta-formulae differ in five segments.
E. flaccidus	Cephalosome of *E. ereimia* sp. nov. wider than long, not longer than wide as in *E. flaccidus*. Two setae on L2_S2_Endo (= Leg 2, Segment 2 of Endopodite) and L3_S2_Endo in *E. ereimia* sp. nov., compared to one in *E. flaccidus*. Furcal rami of *E. ereimia* sp. nov. longer than wide, not as wide as long as in *E. flaccidus*. The spine seta-formulae differ in four segments.
E. inflatipes	Antennules of *E. ereimia* sp. nov. five-segmented, compared to six in *E. inflatipes*. Abdomen of *E. ereimia* sp. nov. four-segmented, compared to three in *E. inflatipes*. Furcal rami of *E. ereimia* sp. nov. not short and not with four setae as in *E. inflatipes*. Fifth leg of *E. ereimia* sp. nov. two-segmented, compared to one in *E. inflatipes*. The spine seta-formulae differ in four segments.
E. lamellifer	Cephalothorax of *E. ereimia* sp. nov. not fused to segment of first leg as in *E. lamellifer*. Antennules of *E. ereimia* sp. nov. five-segmented, compared to six in *E. lamellifer*. No blade-like lamella on second segment of antennae of *E. ereimia* sp. nov. as in *E. lamellifer*. The spine seta-formulae differ in three segments.

E. macrodactylus	Cephalothorax of *E. ereimia* sp. nov. not fused to segment of first leg as in *E. macrodactylus*. Antennules of *E. ereimia* sp. nov. five-segmented, compared to six in *E. macrodactylus*. Anterior region of genital complex of *E. ereimia* sp. nov. not extremely bulged laterally as in *E. macrodactylus*. Overall body shape dissimilar. Fifth leg of *E. ereimia* sp. nov. two-segmented, compared to one in *E. macrodactylus*. The spine seta-formulae differ in nine segments.
E. megacheir	Cephalothorax of *E. ereimia* sp. nov. not quadrangular as in *E. megacheir*. Antennae of *E. ereimia* sp. nov. not with recurved terminal claw as in *E. megacheir*. Thoracic segments of *E. ereimia* sp. nov. laterally not pointing posteriorly. Fifth leg of *E. ereimia* sp. nov. not as extremely small as in *E. megacheir*. Abdomen of *E. ereimia* sp. nov. less than third of total body length, compared to more than third of total body length in *E. megacheir*. Genital segment of *E. ereimia* sp. nov. not oval as in *E. megacheir*. Furcal rami of *E. ereimia* sp. nov. longer than last abdominal segment, compared to equal in *E. megacheir*. No blade-like lamella in second segment of antennae of *E. ereimia* sp. nov. as in *E. megacheir*. Fifth leg of *E. ereimia* sp. nov. two-segmented, compared to one in *E. megacheir*. The spine seta-formulae differ in 10 segments.
E. mirabilis	Cephalosome of *E. ereimia* sp. nov. not the same shape as in *E. mirabilis*. Antennules of *E. ereimia* sp. nov. five-segmented, compared to six in *E. mirabilis*. Fifth leg of *E. ereimia* sp. nov. two-segmented, compared to one in *E. mirabilis*. The spine seta-formulae differ in 11 segments.
E. nodosus	No plumose setae on posterior margin of fourth segment of antennules of *E. ereimia* sp. nov. as in *E. nodosus*. Body of *E. ereimia* sp. nov. not elongate-obovate broadest anteriorly as in *E. nodosus*. Cephalic segment of *E. ereimia* sp. nov. not short as in *E. nodosus*. Antennae of *E. ereimia* sp. nov. not enormous as in *E. nodosus*. Fifth leg of *E. ereimia* sp. nov. present, compared to lacking in *E. nodosus*. The spine seta-formulae differ in seven segments.
E. sarsi	Antennules of *E. ereimia* sp. nov. five-segmented, compared to six in *E. sarsi*. First endopodal segment of antennae of *E. ereimia* sp. nov. not longer than second and no wide depression on second endopodal segment as in *E. sarsi*. Fifth leg of *E. ereimia* sp. nov. two-segmented, compared to one in *E. sarsi*. Egg sacs of *E. ereimia*

	sp. nov. not short as in *E. sarsi*. The spine seta-formulae differ in four segments.
E. briani	Fifth leg of *E. ereimia* sp. nov. two-segmented, compared to one in *E. briani*. Shape of cephalothorax dissimilar. Antennules of *E. ereimia* sp. nov. five-segmented, compared to six in *E. briani*. Abdomen of *E. ereimia* sp. nov. four-segmented, compared to three in *E. briani*. Egg sacs of *E. ereimia* sp. nov. not short and stubby as in *E. briani*. The spine seta-formulae differ in 10 segments.
E. lizae	Cephalothorax of *E. ereimia* sp. nov. not oblong as in *E. lizae*. Antennules of *E. ereimia* sp. nov. five-segmented, compared to six in *E. lizae*. Overall body shape dissimilar. Abdomen of *E. ereimia* sp. nov. four-segmented, compared to three in *E. lizae.* The spine seta-formulae differ in five segments.
E. hypomesi	Segmentation between cephalosome and first pedigerious of *E. ereimia* sp. nov. is prominent, compared to not prominent in *E. hypomesi*. Cephalosome of *E. ereimia* sp. nov. wider than long, compared to longer than wide in *E. hypomesi*. Antennules of *E. ereimia* sp. nov. five-segmented, compared to four in *E. hypomesi*. Antennae of *E. ereimia* sp. nov. not bearing small exopod as in *E. hypomesi*. The spine seta-formulae differ in two segments.
E. caparti	Antennules of *E. ereimia* sp. nov. five-segmented, compared to six in *E. caparti*. First thoracic segment of *E. ereimia* sp. nov. not long as in *E. caparti*. Furcal rami of *E. ereimia* sp. nov. longer than wide, compared to rectangular in *E. caparti*. Fifth leg of *E. ereimia* sp. nov. two-segmented, compared to one in *E. caparti*. The spine seta-formulae differ in 12 segments.
E. parasarsi	First thoracic segment of *E. ereimia* sp. nov. not long as in *E. parasarsi*. Antennules of *E. ereimia* sp. nov. five-segmented, compared to six in *E. parasarsi*. Furcal rami of *E. ereimia* sp. nov. longer than wide, compared to rectangular in *E. parasarsi*. The spine seta-formulae differ in nine segments.
E. parvus	Genital segment of *E. ereimia* sp. nov. not narrowed in posterior third as in *E. parvus*. Furcal rami of *E. ereimia* sp. nov. longer than wide, compared to rectangular in *E. parvus*. Antennules of *E. ereimia* sp. nov. five-segmented, compared to six in *E. parvus*. The spine seta-formulae differ in eleven segments.

E. ilani	Cephalothorax of *E. ereimia* sp. nov. not bullet-shaped as in *E. ilani*. Furcal rami of *E. ereimia* sp. nov. longer than wide, compared to rectangular in *E. ilani*. Antennules of *E. ereimia* sp. nov. five-segmented, compared to six in *E. ilani*. The spine seta-formulae differ in 12 segments.
